# Liposomal Nanocarriers in Non-Alcoholic Fatty Liver Disease: A Systematic Review of Formulation Design, Targeting Strategies, and Therapeutic Outcomes

**DOI:** 10.3390/cimb48090883

**Published:** 2026-08-30

**Authors:** Sahar M. AlMotwaa, Waad A. Al-Otaibi

**Affiliations:** 1 Applied College, Shaqra University, Shaqra 11961, Saudi Arabia; 2 Department of Chemistry, College of Science and Humanities, Shaqra University, Shaqra 11961, Saudi Arabia

**Keywords:** non-alcoholic fatty liver disease (NAFLD), non-alcoholic steatohepatitis (NASH), liposomes, nanomedicine, targeted drug delivery, liver targeting, liposomal nanocarriers, lipid metabolism, oxidative stress, inflammation, hepatic fibrosis, insulin resistance

## Abstract

Non-alcoholic fatty liver disease (NAFLD) affects one-quarter of the global population. The disease may progress from simple steatosis to non-alcoholic steatohepatitis (NASH), fibrosis, and hepatocellular carcinoma. Unfortunately, lifestyle interventions and pharmacotherapies provide limited benefits to NAFLD patients. This systematic review which was conducted according to PRISMA 2020 guidelines, study selection, and data extraction, evaluated preclinical studies published between 1 January 2021 and 4 April 2026 that investigated liposome-based nanocarriers for NAFLD/NASH. Out of 477 records identified, 19 studies met the eligibility criteria and were included in the review. The included studies demonstrated that liposomal formulations prepared using thin-film hydration exhibited characteristics such as particle sizes of 80–160 nm, low polydispersity, zeta potentials of −55 to +35 mV (depending on surface modification), encapsulation efficiencies exceeding 75%, and sustained biphasic release profiles. Mechanistically, these nanocarriers targeted pathways by suppressing de novo lipogenesis through inhibition of FASN and SREBP-1c, enhancement of fatty acid oxidation through activation of AMPK signaling, mitigation of oxidative stress through Nrf2-mediated antioxidant responses, suppression of inflammation via NF-κB and TLR4 signaling, and attenuation of hepatic fibrosis via inhibition of the TGF-β/Smad pathway. Selected formulations also achieved adipose tissue targeting, thereby highlighting their potential to modulate liver–adipose tissue metabolic crosstalk. Despite these promising preclinical findings, clinical translation remains limited by methodological heterogeneity, inconsistent stability assessment, insufficient safety data, absence of clinical trials, biological barriers such as rapid mononuclear phagocyte system clearance, protein corona formation, and restricted penetration through the fibrotic extracellular matrix. Moreover, there are challenges in large-scale manufacturing and batch-to-batch reproducibility. Thus, rigorous toxicological evaluation and well-designed clinical trials are essential for facilitating the clinical translation of liposomal nanomedicines for treatment of liver diseases.

## 1. Introduction

The global prevalence of non-alcoholic fatty liver disease (NAFLD) has risen to approximately 30% and continues to increase, thereby posing a major public health challenge due to its close association with obesity, insulin resistance, type 2 diabetes mellitus, and metabolic syndrome [1]. The disease encompasses a pathological spectrum ranging from simple hepatic steatosis to non-alcoholic steatohepatitis (NASH), which may progress to advanced fibrosis, cirrhosis, liver failure, and hepatocellular carcinoma [2]. Beyond hepatic complications, NAFLD is increasingly recognized as a multisystem disorder which significantly increases the risk of cardiovascular disease, chronic kidney disease, and all-cause mortality [3]. Non-alcoholic fatty liver disease (NAFLD) develops through complex interactions among metabolic, inflammatory, genetic, and environmental factors, rather than a single pathogenic mechanism [4]. According to the widely accepted “multiple-hit” theory, insulin resistance, dysregulated lipid metabolism, oxidative stress, mitochondrial dysfunction, endoplasmic reticulum stress, chronic inflammation, gut microbiota dysbiosis, and progressive hepatic fibrosis all contribute to hepatocellular injury and disease progression [5].

Lifestyle modification remains a cornerstone of NAFLD management. However, achieving and sustaining clinically meaningful weight loss and long-term metabolic improvement are challenging for many patients [2,6]. Although several pharmacological therapies have shown encouraging therapeutic potential, no single treatment has demonstrated consistent efficacy across all stages of NAFLD or NASH [7]. The clinical development of many investigational therapeutics is further complicated by physicochemical, pharmacokinetic, and safety limitations. These constraints comprise poor aqueous solubility, low oral bioavailability, rapid systemic clearance, inadequate hepatic delivery, and dose-limiting toxicity, all of which compromise therapeutic efficacy and hinder clinical translation [8]. Accordingly, advanced drug delivery systems have emerged as promising strategies for improving drug solubility and stability, optimizing pharmacokinetic behaviour, enhancing hepatic drug delivery, and minimizing off-target toxicity.

Recent advances in nanomedicine have provided innovative solutions to these challenges through nanoscale drug delivery systems capable of improving drug stability and enhancing pharmacokinetic properties [9]. Among various nanocarriers, liposomes have garnered considerable attention owing to their high biocompatibility, biodegradability, formulation flexibility, and established safety record. The phospholipid bilayer structure of liposomes allows for simultaneous encapsulation of both hydrophilic and lipophilic therapeutic compounds while protecting them from premature degradation [10]. Moreover, advances in liposome engineering such as PEGylation, polymer coating, ligand-mediated targeting, and stimuli-responsive systems, have improved formulation stability, prolonged circulation time, and enhanced targeting efficiency. These qualities have established liposomes as one of the most extensively investigated nanocarriers for liver-directed drug delivery [10,11]. Due to its distinct anatomical and physiological features, the liver is an ideal target for nanoparticle-based medication delivery systems. Its wide circulatory network, fenestrated sinusoidal endothelium, and varied resident cell populations make it easy for hepatic cells to accumulate and absorb suitably engineered nanocarriers [12].

**Figure 1:**
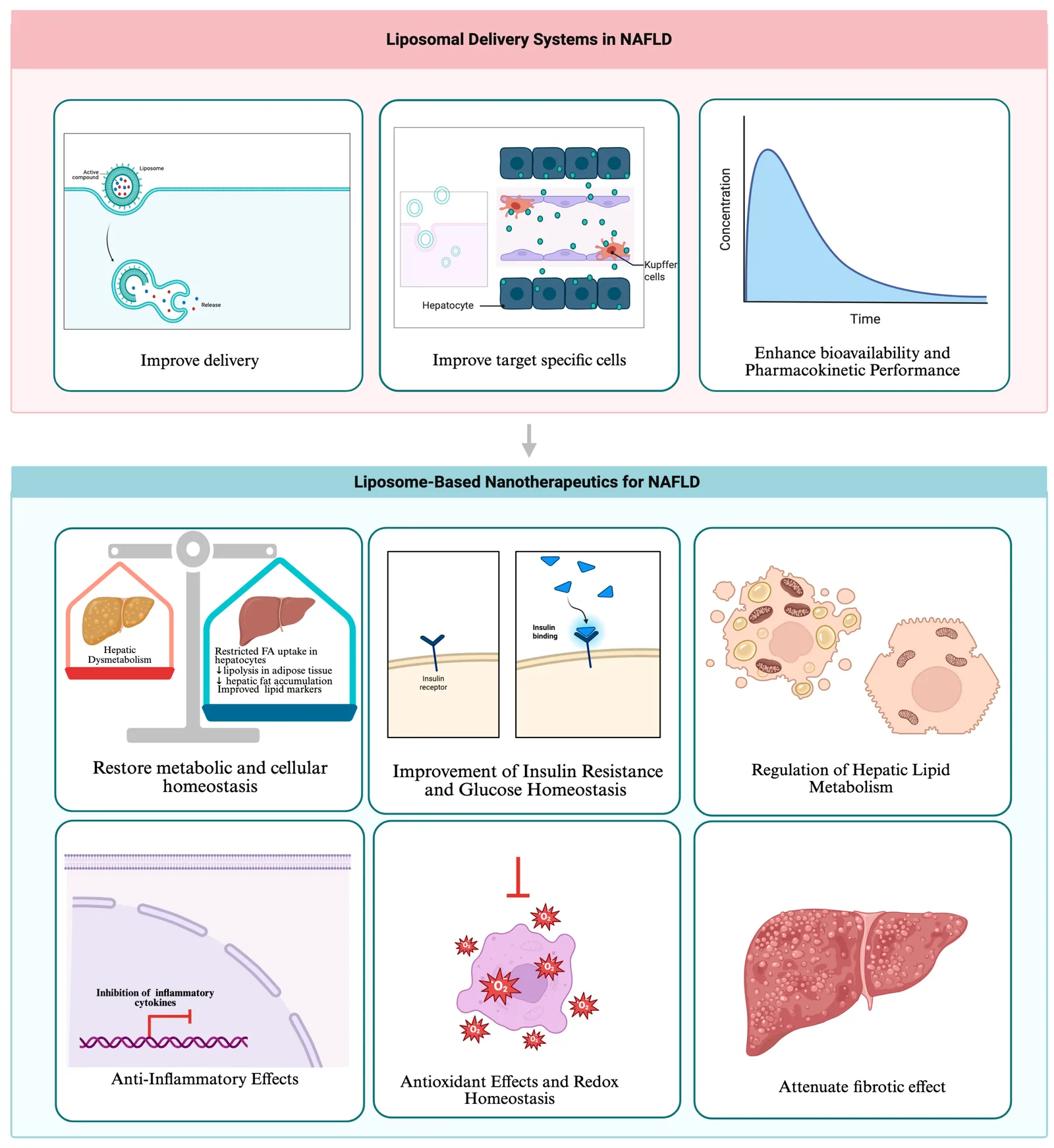
Overview of the therapeutic advantages of liposome-based delivery systems in NAFLD. ↓, decrease. Created in BioRender. M, S. (2026) https://BioRender.com/vzo0uxw (accessed on 29 June 2026).

A wide range of therapeutic agents such as bioactive natural chemicals, synthetic small-molecule medicines, peptides, proteins, hormones, and nucleic acid-based therapies, have been encapsulated due to the significant flexibilities of liposomal formulations [10,12,13]. Preclinical data have shown that these systems improve the delivery of therapeutic agents to the liver, thereby halting development of hepatic fibrosis by reducing hepatic steatosis, restoring lipid homeostasis, improving insulin sensitivity, lowering oxidative stress, and decreasing inflammatory signaling [14,15,16]. Figure 1 is a summary of the proposed relationships among key structural characteristics of liposomal drug delivery systems, their design strategies, and the corresponding therapeutic mechanisms involved in NAFLD. This conceptual framework provides an overview of how formulation properties may influence hepatic targeting and therapeutic outcomes, and it establishes the rationale for the present systematic review.

However, the clinical translation of liposomal nanocarriers remains limited by fragmented and heterogeneous preclinical evidence. Existing studies differ substantially in formulation composition, particle architecture, surface engineering, targeting strategies, physicochemical characterization, experimental models, and outcome measures. These differences constitute constraints to cross-study comparisons, and obscure the impact of specific design features on hepatic targeting and therapeutic efficacy. Furthermore, no previous review comprehensively integrated formulation characteristics, encapsulated therapeutics, targeting strategies, mechanistic pathways, and therapeutic outcomes, within a unified analytical framework. Addressing these limitations is essential for identifying evidence-based design principles, strengthening interpretation of preclinical findings, and accelerating the clinical translation of liposome-based nanomedicines.

Therefore, in this systematic review, a comprehensive evaluation was conducted on extant preclinical evidence of liposome-based drug delivery systems for the treatment of NAFLD and NASH. Specifically, the review examined how liposomal formulation design, encapsulated therapeutic agents, and targeting strategies influenced hepatic drug delivery and therapeutic outcomes, with respect to lipid metabolism, insulin resistance, oxidative stress, inflammation, and hepatic fibrosis. Furthermore, the review identified current limitations in formulation design and experimental methodologies and highlighted future research priorities for facilitating the rational development and clinical translation of liposome-based therapeutics for NAFLD and NASH.

## 2. Materials and Methods

### 2.1. Design of the Study

This systematic review was performed to comprehensively evaluate the therapeutic applications of liposomal nanocarriers in the treatment of NAFLD and NASH. A literature search was conducted on 4 April 2026. A predetermined two-stage research selection procedure was utilized, in the light of significant variations in nanoparticle nomenclature found in published works. All nanoparticle-based medicinal trials were first identified using a purposefully broad search technique, instead of limiting initial database search specially to liposomal systems. Then, predetermined liposomal inclusion criteria were implemented after eligible studies were categorized based on the delivery platform and nanoparticle composition. This approach was in strict adherence to the principal goal of the review. Moreover, it reduced the possibility of missing pertinent liposomal research due to inconsistent indexing and differences in nanoparticle terminologies. The selection process was designed and conducted in line with the Preferred Reporting Items for the Systematic Reviews and Meta-Analysis (PRISMA 2020) guidelines [17]. This systematic review has been registered in the Open Science Framework (OSF), Center for Open Science, Washington, DC, USA, under registration number: jr9z4 (https://osf.io/jr9z4, accessed on 19 August 2026).

### 2.2. Methods Used for Literature Search

A thorough systematic literature search was carried out using PubMed, Scopus, and Web of Science core collection (WoS), for the purpose of identifying research on nanoparticle-based therapeutic approaches for NAFLD and NASH. Three pre-established idea groups were incorporated into the search strategy: terms connected to diseases, terms related to nanotechnology that describe drug delivery methods based on nanoparticles, and experimental approaches that identify in vitro, in vivo and clinical therapeutic investigations.

A purposefully wide search approach was used to increase search sensitivity. All nanoparticle-based therapeutic studies were first retrieved and then subjected to manual nanoparticle classification during the eligibility assessment stage. This approach allowed us to capture all relevant studies, including those that may have been inconsistently indexed or lacked explicit liposome-related terms in their titles or abstracts. While maintaining the same basic framework, separate database-specific search algorithms were created for PubMed, Scopus and WoS. To enable consistent retrieval across databases, search syntax was modified in accordance with the indexing requirements of each database [Title/Abstract for PubMed, TITLE-ABS-KEY for Scopus, and Topic (TS) for WoS]. The literature search was performed on 4 April 2026. The searches in all databases were confined to English-language papers published between 1 January 2021 and 4 April 2026. The complete database-specific search strategies are shown in Table 1. All search syntax strings are presented exactly as executed in each database to ensure full reproducibility.

### 2.3. Manual Classification of Nanoparticles as an Additional Eligibility Criterion

Since every nanoparticle-based medicinal research was intentionally included in the first search technique, an additional specified eligibility criterion was subsequently applied via manual nanoparticle classification. Using nanoparticle composition and delivery platform, the nanoparticles were independently classified by W. A. and S. M., instead of using author-defined terms.

The categories into which the therapeutically relevant nanoparticle research were divided were polymeric nanoparticles, lipid-based nanoparticles, nanoparticles, hybrid nanoparticles, and liposomal formulations. The predetermined inclusion criteria of the review were satisfied only by liposome-based delivery systems such as conventional liposomes, PEGylated liposomes, ligand-functionalized liposomes, multilamellar liposomes, and other modified liposomal formulations.

### 2.4. Collection of Data

The reviewers (S. M. and W. A.) extracted the data independently using a standardized extraction form prepared before the study was started. The extracted data were compared to the original full-text articles and, where available, with other resources to ensure accuracy and completeness. For each eligible study, data were carefully extracted, with respect to formulation platform, therapeutic cargo, lipid composition, surface modification or functionalization, targeting strategy, experimental model, route of administration, dose, treatment duration, biodistribution, cellular uptake, safety and biocompatibility, therapeutic outcomes, and proposed mechanisms of action. Structured comparison analysis was used to qualitatively synthesize the data. Methodical comparison was carried out on the included studies, with respect to formulation design, lipid content, surface engineering techniques, targeting mechanisms, biodistribution profiles, therapeutic efficacy, safety results, and suggested molecular pathways.

## 3. Results and Discussion

### 3.1. Study Selection

**Figure 2:**
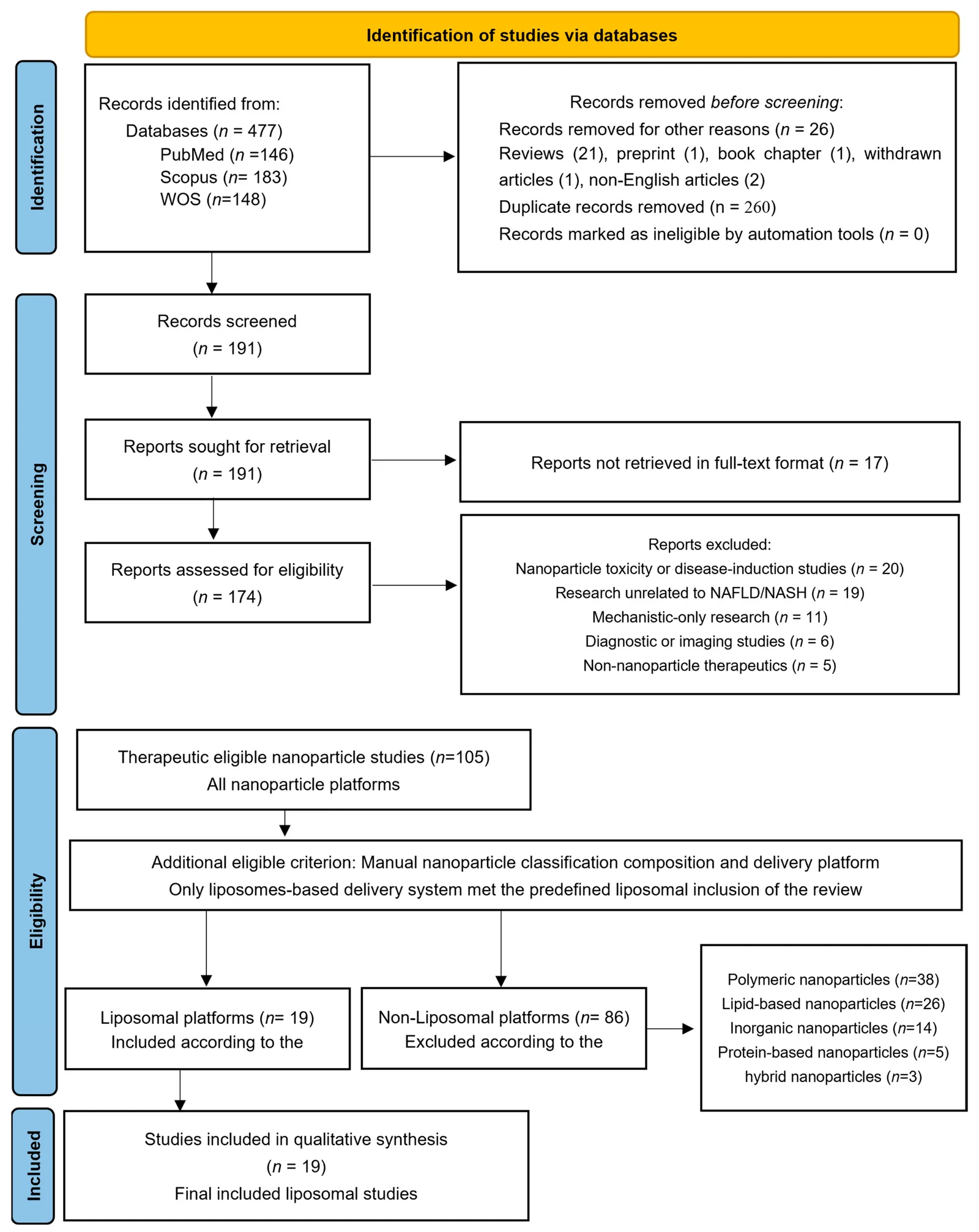
Prisma flow diagram for the current systematic review. Source: [17]. This work was licensed under CC By 4.0. To view a copy of this license, visit https://creativecommons.org/licenses/by/4.0/ (accessed on 29 June 2026).

The study selection process is summarized in Figure 2. The search from databases yielded a total of 477 studies comprising 146 PubMed, 183 Scopus, and 148 WoS. The studies were independently selected by two reviewers (S. M. and W. A.) Twenty-six (26) entries consisting of 21 review articles, 1 preprint, 1 book chapter, 1 withdrawn article, and 2 non-English publications, were excluded prior to screening, because they did not meet the pre-determined publication eligibility criteria. The titles and abstracts of the remaining 191 publications were screened and considered as potentially relevant studies eligible for full-text evaluation. However, 17 out of the 191 reports were not accessible, leaving 174 full-text publications for eligibility evaluation. The full-text articles were independently assessed in accordance with predetermined inclusion and exclusion criteria. Studies that examined nanoparticle-based therapeutic approaches for NAFLD and NASH, research that reported novel therapeutic results using in vitro, in vivo, or clinical models, and studies accessible as full text papers published in English, were all deemed eligible. Based on these criteria, a total of 69 studies were excluded, resulting in 105 therapeutically eligible nanoparticle studies. Subsequent manual nanoparticle classification according to nanoparticle composition and delivery platform led to the identification of 19 studies which employed liposomal delivery systems that fulfilled the predefined liposomal inclusion criterion. Thus, 19 studies were retained for the final qualitative synthesis, whereas the remaining 86 studies based on non-liposomal nanocarriers, were excluded.

### 3.2. Methods Used for Preparation of Liposomal Systems in NAFLD and NASH Studies

**Figure 3:**
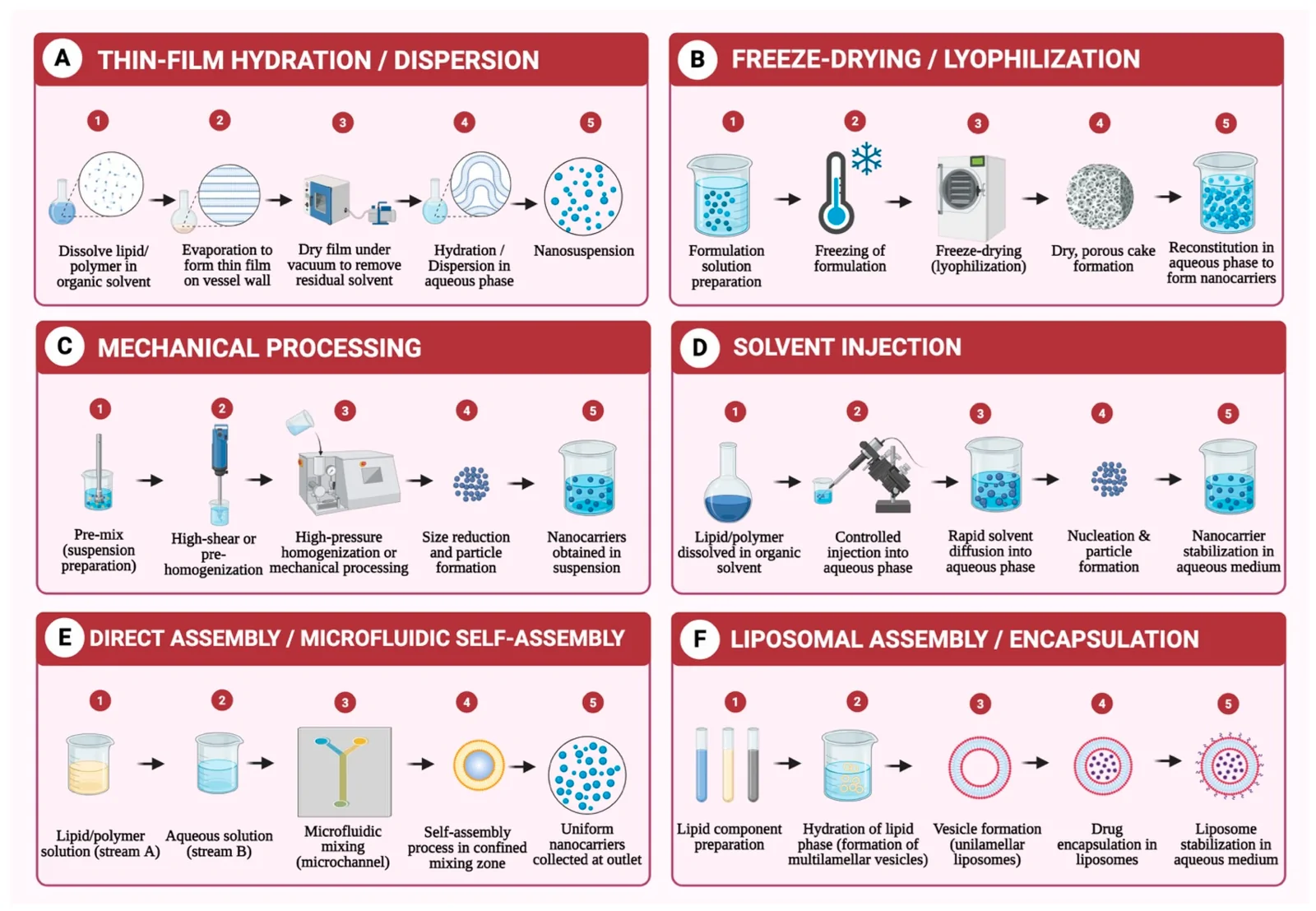
Classification of Liposome Preparation Methods in NAFLD and NASH Research. Created in BioRender. M, S. (2026) https://BioRender.com/p1ii1nk (accessed on 29 June 2026).

Thin film-based methods were the most widely applied of these strategies, as shown in Figure 3. Thin-film-based techniques such as thin-film hydration and thin-film dispersion are often used for making most liposomal formulations. Indeed, DEAE-DEX@SDBS [18], liposomal silybin [14], silibinin-loaded liposomes (Sil-Lip) [16], and chitosan-coated lutein liposomes (Lu-lip-LC and Lu-lip-HC) [19] were all prepared via thin-film dispersion. Moreover, thin film-based procedures were applied in the preparation of PEGylated vitexin liposomes (PEG-VLPs) [20], dasatinib/quercetin liposomes (D+Q liposomes) targeting white adipose tissue [21], dual-targeted sodium acetate liposomes (NaA@SC/MAN-LPs) [22], glucagon-modified triiodothyronine liposomes (GCG-lipo/T3) [23], chrysin-loaded nanoliposomes (CH-NL) [24], aptamer-functionalized allicin liposomes (LAA) [25], and the CD47-modified hybrid liposomal platform [26]. Surface-engineering techniques such as hyaluronic acid functionalization in ROS-responsive liposomes (RLLs) [15], ligand conjugation, PEGylation, polymer coating, remote loading, and immune-evasive alterations, were also incorporated in a number of formulations. Despite these differences, the basic manufacturing process for all systems was the same: lipid components were dissolved in organic solvents, evaporated to create a thin lipid film, and then hydrated to create vesicular nanostructures. Sonication, extrusion, filtration, and homogenization are examples of additional processing techniques that were frequently used to enhance formulation quality and particle-size uniformity.

On the other hand, several studies adopted alternative techniques for liposome preparation. These techniques involved liposomal assembly and encapsulation, followed by PEGylation and adipose-homing peptide conjugation [27], freeze-drying (lyophilic freezing) [28], dehydration-rehydration method [29], ether injection technique [30], microfluidic self-assembly [31], high-pressure homogenization [32], and ultrasonic fragmentation followed by electrostatic deposition [33]. Overall, the diverse preparation methods reflect ongoing efforts to optimize liposome properties such as encapsulation efficiency, stability, biodistribution, and targeted drug delivery.

### 3.3. Liposomal Properties in Experimental NAFLD/NASH Models

#### 3.3.1. Physicochemical, Colloidal, and Morphological Characteristics

The behaviors of liposomal systems in the included studies were governed by factors such as particle size, polydispersity index (PDI), surface charge, morphology, encapsulation efficiency (EE), drug loading (DL), release behavior, and storage stability, as indicated in Table S1. These factors eventually influenced the biological performance of liposomal systems. The combined influence of these elements shape circulatory dynamics, tissue distribution, and cellular interaction patterns [34]. Most liposomal particle diameters ranged from 80 to160 nm. This range is often linked to beneficial colloidal behavior, extended circulation, decreased phagocytic clearance, and increased hepatic exposure [35]. In addition, it offers a good compromise between systemic stability and cellular absorption [36]. Only three studies were reported with droplet diameters above 200 nm [19,20,30]. In addition, slightly larger nanocarriers resulted in longer gastrointestinal residency and more robust contact with the mucus layer in oral administration scenarios, which indirectly improved hepatic delivery [37,38].

In terms of particle size distribution, particle sizes in the sub-200 nm range were associated with narrow size distributions typically < 0.30 in nanosystems such as GCG-lipo/T3 [23], Gal-LNP-RSV [31], NaA@SC/MAN-LPs [22], aptamer-functionalized allicin liposome (LAA) [25] and DEAE-DEX@SDBS [18], indicating a comparatively monodisperse population. Similar patterns were seen in number of traditional liposomal formulations, for example, CH-NL [24] and Sil-Lip [16], which showed particle sizes between 100 and 135 nm. Vesicle size was enhanced by coating with chitosan [19] and hyaluronic acid (HA) [15] without sacrificing size uniformity.

Interestingly, mannose functionalization in M-Lip/PTM caused only slight size changes, when compared to the unmodified system [29,39], indicating that ligand conjugation was accomplished without significantly altering the structural organization of the liposomal carrier. On the other hand, some studies did not report PDI values, while some others reported values ≥ 0.3. Despite variations in composition and surface engineering techniques, most formulations maintained a comparatively homogeneous particle population, as seen in the predominance of low PDI values and nanoscale particle sizes across the included investigations.

Zeta potential (ZP) values varied significantly among the studies, indicating variations in surface engineering techniques and formulation composition. A number of formulations such as SIM-LipoNPs [28], CH-NL [24], D+Q liposomes [21], hybrid liposomal platform [26], GCG-lipo/T3 [23], and hyaluronic acid-coated ROS-responsive liposomes (RLLs) [15], showed clearly negative surface charges, typically between −20 and −55 mV. The ionized carboxyl groups on the hyaluronic acid coatings in the RLLs were responsible for the negative charge [40]. The charge properties were often changed by surface functionalization, which also provided indirect proof of effective surface modification. Particularly, aptamer conjugation changed the surface of allicin-loaded liposomes from almost neutral to noticeably negative [25], suggesting that the negatively charged aptamer moieties were successfully incorporated. A similar behavior was seen in spirulina peptide-loaded liposomes, where the surface charge changed from negative to positive after chitosan coating, whereas peptide loading decreased the amplitude of the negative charge [32]. Chitosan-coated lutein liposomes exhibited a similar charge reversal, changing from a highly negative surface to moderately positive values after coating [19]. This change is often seen as a sign of good surface deposition, and it is consistent with the cationic nature of chitosan [41].

On the other hand, formulations such as NaA@SC/MAN-LPs [22], gum Arabic-coated anthocyanin liposomes [33], and mannose-modified platensimycin liposomes [29], showed positive zeta potential values between +10 and +35 mV. Positively charged nanocarriers interface more easily with negatively charged biological membranes and mucus layers, resulting in improvement of cellular uptake and mucosal retention. However, under some physiological circumstances, excessive positive charge may potentially accelerate clearance and increase non-specific interactions with biological components [42]. Other systems showed more subtle charge modulation, following DEAE-DEX coating [18]. The ZP-loaded formula gradually changed from a slightly positive value to a mildly negative one before nearing neutrality, suggesting the generation of a multilayer structure. Similarly, PEGylation produced a nearly neutral surface by reducing the strength of the negative charge in vitexin liposomes [20]. This finding is in line with the well-known charge-shielding action of PEG chains, which improves steric stabilization and conceal surface charges [35]. Silybin-loaded liposomes retained a near-neutral-to-mildly negative surface charge [14], while the addition of the PTP ligand changed the surface charge of the adipose-targeted PLT3 formulation from slightly negative to slightly positive values [27].

Regarding integrity, analysis with TEM, SEM, or AFM revealed that most liposomal formulations were primarily spherical or nearly spherical in morphology. This structural consistency was evident in multifunctional nanocarriers and conventional liposomes such as PEGylated, polysaccharide-coated, peptide-functionalized, and aptamer-modified systems. These comprised PEG-VLPs [20], PLT3 [27], SLB-liposomes [14], SIM-LipoNPs [28], D+Q liposomes [21], NaA@SC/MAN-LPs [22], Lip/PTM and M-Lip/PTM [29], CH-NL [24], Lip-Lyco [30], Lu-lip formulations [19], RLLs [15], Gal-LNP-RSV [31], SP-LP/SP-Ch-LP [32], LAA [25], and the hybrid liposomal platform [26], Each of these systems maintained a spherical vesicular architecture with relatively uniform size distribution.

However, subtle variations were noted during surface engineering, despite the general physical similarities. In DEAE-DEX@SDBS [18] and Sil-Lip [16], the vesicles were surrounded by diffuse or fluffy peripheral layers which are indicative of surface alteration mediated by polymers or coatings. On the other hand, following polysaccharide stabilization, gum Arabic-coated anthocyanin liposomes (ACNs-LNP) [33] displayed compact and tightly packed nanostructures, whereas NaA@SC/MAN-LPs [22] had a homogeneous distribution with no discernible aggregation. Additionally, despite significant surface alteration, the hybrid liposomal formulations [26] were described as uniform unilamellar vesicles, demonstrating good structural retention. Overall, the available morphological data indicated that most of the surface-engineering techniques changed the interfacial properties of the nanocarriers without affecting their basic vesicular structures. The maintenance of spherical shape in the various formulations may promote the usefulness of liposomal platforms for NAFLD/NASH drug delivery applications by promoting colloidal stability, repeatability, and effective biological interactions.

#### 3.3.2. Drug Loading and Encapsulation Efficiency

Most formulations had EE values above 75%, indicating effective drug entrapment, regardless of variations in cargo, targeting ligands, or surface coatings [14,16,20,21,22,23,24,26,30,31]. Aptamer-functionalized liposomes [25] were among the few formulations that revealed EE values between 50% and 75%, while only conventional and mannose-modified platensimycin liposomes [29] showed values below 50%. Drug loading (DL), which varied from about 0.5 to 33%, was reported less frequently than encapsulation efficiency. While most formulations showed DL values below 10%, for example, conventional and mannose-modified platensimycin liposomes [29] (which had the lowest reported DL), dual-targeted sodium acetate liposomes [22] had the highest DL value, followed by chrysin-loaded nanoliposomes [24] and hybrid liposomal platform [26]. This variation implies that high drug loading is not always correlated with high encapsulation efficiency. While retaining comparatively low loading capacities, some of the formulations attained encapsulation efficiencies greater than 85% [24,26].

Encapsulation performance was typically maintained or improved by surface modification. Polymer-coated and functionalized liposomes such as chitosan-, gum Arabic-, aptamer-, PEG-and ligand-modified systems, consistently showed moderate-to-high EE values, suggesting that surface engineering improved vesicle stability and drug retention, rather than negatively impacting drug entrapment [19,20,25,33]. However, a number of studies such as those based on dextran-coated bilosomes [18], adipose-targeted T3 liposomes [27], simvastatin-loaded nanoliposomes [28], and hyaluronic acid-coated ROS-responsive liposomes [15], did not provide data on EE or DL. Furthermore, a study that did not provide baseline encapsulation values showed changes in EE during storage [32]. Inconsistent reporting in some studies made it difficult to carry out direct comparison of formulation performances. This emphasizes the need for more thorough characterization in subsequent research.

The capacity of liposomal bilayers to hold both hydrophilic and lipophilic compounds, as well as the role of surface modification techniques that improve vesicle integrity and lessen early drug leakage, may be responsible for the preponderance of encapsulation efficiency values above 75%. Drug retention during formulation and storage may be enhanced by stabilizing the lipid bilayer using coating materials and targeted ligands. On the other hand, lower encapsulation efficiencies may result from factors such as drug diffusion during preparation, restricted compatibility between the encapsulated chemical and the lipid matrix, and unfavorable drug–lipid interactions [43]. Furthermore, drug loading is affected by encapsulation efficiency and the quantity of lipid needed to achieve stable vesicle formation. Therefore, the observed variabilities in drug loading capacity probably reflects variations in drug physicochemical properties, lipid composition, and formulation techniques [44].

#### 3.3.3. Kinetic Profiles and Drug Release Behavior

There were variations in drug release characteristics of the liposomal formulations described in the included studies, with most formulations displaying a prolonged or regulated release pattern. Formulation composition, cargo properties, and surface modifications with polymer coatings and targeting ligands, which impact membrane permeability and drug retention, seemed to have influenced variations in release kinetics. The PEGylated vitexin liposomes released approximately 48% of the loaded medication over 72 h [20] whereas, among the prolonged-release systems, GCG-lipo/T3 achieved nearly 70% release after 75–82 h [23]. On the other hand, platensimycin liposomes reached about 90% release within 24 h [29], whereas D+Q liposomes released almost of the encapsulated quercetin and more than 80% of dasatinib within ~50 h [21]. This suggests that formulation composition and drug–lipid interactions significantly affect release behavior.

Modified release kinetics were often linked to surface alteration. Hyaluronic acid-coated ROS-responsive liposomes demonstrated ROS-triggered drug release, in keeping with their intended use in oxidative microenvironments [15]. Similarly, in both lutein-loaded liposomes and spirulina peptide-loaded nanoliposomes, chitosan coating inhibited drug release. After 16 h, drug release at pH 7.4 in lutein formulations dropped from 12.74% in uncoated liposomes to 11.33 and 8.39% in formulations coated with low and high chitosan, respectively [19]. Similarly, when compared to free peptide, chitosan-coated spirulina nanoliposomes decreased peptide release from 42.72 to 22.86% after 4 h, and from >84.05 to ~60.07% after 24 h [32]. These decreases might be due to the formation of chitosan-derived polymeric layer around the liposomal surface. This layer acted as an extra diffusion barrier which lengthened the diffusion path, and limited drug penetration into the release medium, thereby supporting a more sustained release profile [44].

About 45–50% of the silibinin in the hybrid liposomal system was released in less than 24 h, suggesting a moderate sustained-release profile [26]. The formulation Gal-LNP-RSV showed a more sustained release pattern than free RSV, with cumulative release approaching ~70% after about 36 h [31]. At pH 1.2, DEAE-DEX@LSDBC showed an initial burst release of berberine, followed by a continuous release that reached approximately 50% at 24 h, while curcumin release stayed below 20% at 24 h [18]. Generally, most formulations showed a biphasic release pattern characterized by a slower, sustained phase after an initial burst release phase. While the second phase was primarily controlled by diffusion through the liposomal matrix or membrane, the first phase was probably due to the release of surface-associated drug [40]. However, variations in release-testing conditions such as medium composition, pH, temperature, and analytical techniques, influenced the outcomes of direct comparison among studies.

#### 3.3.4. Colloidal Robustness and Storage Stability

Although stabilization techniques were similar among some studies, the reported stability-related data were inconsistent across the included studies. For DEAE-DEX@SDBS [18], Lip-Lyco [30], Lu-lip-LC/Lu-lip-HC [19], and aptamer-functionalized allicin liposomes [25], the most investigated stability condition was refrigerated storage at 4 °C. Furthermore, certain formulations such as SIM-LipoNPs [28], GCG-lipo/T3 [23], and Gal-LNP-RSV [31], were lyophilized and kept at −20 °C for long-term preservation. By reducing water-mediated destabilization processes, freeze-drying was frequently used to lower hydrolytic breakdown and lipid peroxidation, thereby enhancing the storage stability of the liposomal systems [40,45]. Surface engineering may have led to the improved formulation stability reported in some of the studies. The steric stabilization of PEG chains which reduces particle aggregation and inter-vesicular contacts, might account for the enhanced colloidal stability shown by the PEGylated vitexin liposomes [20]. Similarly, gum Arabic-coated anthocyanin liposomes showed improved gastrointestinal and thermal stability [33], possibly because the lipid bilayer was protected from environmental stress and digestive conditions by a layer of polysaccharides [40,46]. Similar findings were documented for formulations coated with chitosan [19,32], where the polymer coating most likely reinforced the interfacial barrier encircling the vesicles, thereby minimizing particle destabilization and reducing leakage of encapsulated material.

It was observed that Gal-LNP-RSV, one of the formulations subjected to quantitative stability tests, maintained its particle size and PDI for 12 days in a medium containing 10% FBS [31]. This behavior suggests resistance to protein-induced aggregation and preservation of colloidal integrity in physiologically relevant environments. Aptamer-functionalized allicin liposomes were stable for 4 weeks at 4 °C, whereas the aptamer was stable for 24 h in 10% FBS medium [25]. In the same vein, the hybrid liposomal platform demonstrated minimal particle size variation at 4 °C for a minimum of 5 days [26], suggesting adequate short-term colloidal stability under cold conditions. On the other hand, the studies on PLT3 [27], D+Q liposomes [21], Lip/PTM and M-Lip/PTM [29], CH-NL [24], and RLLs [15], either only described stability qualitatively, or did not provide data on storage conditions and long-term stability evaluations. This discrepancy in reporting made it difficult to compare formulations directly, and it emphasizes the need for more uniform stability-testing procedures, especially for systems meant for clinical translation and long-term storage. Moreover, cross-study comparisons were restricted by short-term and non-standardized stability assessments. Interestingly, favorable physicochemical characteristics did not necessarily result in better therapeutic outcomes; these outcomes also depended on stage of the illness, as well as drug-related factors such as biodistribution, pharmacokinetics, and target accessibility.

### 3.4. Liposomal Delivery Strategies for Enhanced Hepatic Drug Targeting

#### 3.4.1. Conventional and Polymer-Coated Liposomal Systems

In some of the included studies, the researchers sought to improve the pharmacokinetic performance of liposomal formulations by increasing oral absorption and systemic bioavailability. It is known that these techniques promote vesicle stability during gastrointestinal transit, and increase intestinal absorption and systemic drug exposure after oral delivery. Among the polymer-coated formulations, dextran-coated multilayer bilosomes produced the most improvement after oral administration. These bilosomes enhanced the mucus and cell penetrations of Caco-2 [18], and increased the area under the concentration-time curve by more than ten folds. As a result, the formulations produced the greatest hepatic accumulation among the reported studies, and the oral bioavailability values for curcumin and berberine increased by 13 and 34 times, respectively. Similarly, the surface modification of chitosan-coated liposomes enhanced the uptake of lutein by HepG2 and HUVEC cells [19]. In addition, gum Arabic-coated liposomal nanoparticles improved the anthocyanin absorption and antioxidant status of HepG2 cells [33]. Chitosan-coated spirulina peptide liposomes showed better therapeutic efficacy than the free peptide formulation, although direct biodistribution and cellular uptake analyses were not performed [32]. Extant evidence indicates that polysaccharide-based surface coatings enhance the oral delivery performance of liposomal formulations through a variety of complementary mechanisms. However, this conclusion is based on a comparatively small number of studies using various formulations and experimental models. Polysaccharide-based surface coatings improve mucoadhesion, extend intestinal residence time, lessen early breakdown of encapsulated drugs, and probably facilitate intestinal absorption into the portal circulation, in addition to shielding vesicles from the harsh gastrointestinal environment. Nevertheless, physicochemical characteristics such as surface charge, hydrophilicity, particle size, and coating density exert significant impacts on the performance of these coatings. Access to the intestinal epithelium may be restricted by strong interactions with the mucus layer, which may even prevent deep mucus penetration [47]. Therefore, the higher hepatic exposure attained following oral treatment should be primarily regarded as a result of enhanced gastrointestinal absorption and portal delivery, rather than conclusive evidence of receptor-mediated liver targeting.

Conventional, non-targeted liposomal formulations also showed changes in pharmacokinetic behavior comparable with those of targeted formulations. While chrysin-loaded liposomes resulted in 2.04- and 3.32-fold enhancements in serum drug concentrations and hepatic accumulation, respectively [24], silibinin-loaded liposomes increased the relative oral bioavailability of silibinin by 9.45 folds, when compared to the free drug [16]. Traditional lycopene-loaded liposomes increased therapeutic efficacy in an experimental NAFLD study in which tissue distribution or cellular uptake was not assessed [30]. Similarly, in another study without quantitative biodistribution data, liposomal silybin improved drug delivery [14]. Despite the lack of information on quantitative biodistribution, PEGylated vitexin liposomes decreased histopathological damage in the kidney and spleen, when compared to free vitexin, indicating better systemic distribution and reduced off-target toxicity [20]. In contrast, one study did not present data on in vivo biodistribution or targeting efficiency, because it was restricted to in vitro characterization of simvastatin-loaded liposomes [28].

When combined, these results show that both polymer-assisted surface engineering and traditional liposomal encapsulation significantly increased the pharmacokinetic performance and oral bioavailability of poorly soluble bioactive substances. However, increased hepatic medication exposure by itself should not be taken as evidence of liver-specific targeting. Rather than being caused by targeting mechanisms, passive hepatic accumulation may also be caused by physiological features of the liver, such as fenestrated sinusoidal endothelium, high blood perfusion, and interactions with the mononuclear phagocyte system. Many biological mechanisms such as intestinal absorption efficiency, protein corona formation, uptake by the mononuclear phagocyte system, and the physicochemical properties of the nanocarrier itself, regulate the in vivo fate of orally delivered nanoparticles [11,12]. Therefore, quantitative tissue biodistribution studies backed by mechanistic validation such as receptor-blocking experiments or cellular uptake investigations, are necessary for provision of strong evidence of liver-specific targeting. These investigations were not done in the studies included in this review [46]. Such evidence is especially crucial for the clinical translation of oral nanocarrier systems, and for differentiating better systemic exposure from real liver-specific targeting.

#### 3.4.2. Active Liposomal Targeting Strategies

A second set of experiments used active targeting techniques to facilitate selective distribution to particular tissues or cell populations through ligand–receptor interactions, instead of traditional liposomal formulations that mainly improve pharmacokinetic performance. These strategies were aimed at achieving tissue selectivity, increasing intracellular absorption, and decreasing off-target distribution, through functionalization of the liposomal surface with ligands capable of identifying overexpressed receptors in sick tissues. One of these strategies involved the design of adipose tissue-targeting systems to take advantage of the metabolic role this tissue plays in the development of NAFLD. Peptide-functionalized T3 liposomes showed altered biodistribution patterns consistent with potential adipose tissue targeting to the liver, heart, kidney, brain, lung, and skeletal muscle while preferentially accumulating in inguinal white, epididymal white, and brown adipose tissues [27]. Similarly, P3-functionalized dasatinib/quercetin liposomes showed enhanced accumulation in white adipose tissue compared to their non-targeted counterparts [21]. These results imply that modification of the function of adipose tissue may be a useful but indirect method of enhancing hepatic metabolic balance. Several receptor-specific surface changes were used to effect direct hepatic targeting. A study which took advantage of mannose receptor-mediated absorption demonstrated that mannose-functionalized liposomes were associated with increased liver accumulation [29]. In addition, relative to free sodium acetate, NaA@SC/MAN-LPs produced a 3.75-fold increase in hepatic acetate accumulation, an effect that was supported by SC and MAN competition assays confirming receptor involvement [22]. Similarly, liver-directed administration and therapeutic efficiency were enhanced by mannose-modified platensimycin liposomes [29]. Glucagon-conjugated liposomes produced higher hepatic fluorescence intensity compared to non-targeted liposomes, resulting in enhanced hepatic enrichment of triiodothyronine [23]. Similarly, galactose-modified lipid nanoparticles targeting the asialoglycoprotein receptor (ASGPR) produced 3.5-fold increase in cellular absorption as well as a 3.9-old improvement in hepatic fluorescence intensity [31]. This data represents the strongest mechanistic sign of receptor-mediated targeting with a 56.7% decrease in cellular uptake after galactose competition. However, direct comparisons across various ligand systems were challenging because none of the included studies rigorously measured effectiveness of liver-targeting using standardized metrics.

The range of active targeting techniques was further expanded by other receptor-targeted methods. Hyaluronic acid-coated ROS-responsive liposomes showed enhanced uptake by AML-12 and LX-2 cells, an effect that was inhibited by HA competition, supporting CD44-mediated interactions [15]. Similarly, aptamer-functionalized allicin liposomes showed enhanced hepatic accumulation and higher absorption in NAFLD-associated cells, relative to HepG2 cells, although receptor competition assays were not performed to confirm CD36 specificity. Taken as a whole, these investigations demonstrate the adaptability of ligand-mediated surface engineering in guiding liposomal nanocarriers toward metabolically significant regions via unique biological recognition pathways [25].

While ligand-functionalized formulations often provided stronger mechanistic rationale than passive systems, enhanced cellular uptake or improved biodistribution alone does not confirm receptor-mediated targeting [11,47]. Robust confirmation requires receptor-blocking studies or quantitative biodistribution analyses demonstrating receptor-dependent accumulation [11]. Among the included studies, only three [15,22,31] fulfilled these criteria, whereas the remaining ligand-modified formulations [15,21,23,25,27,29] were classified as putative targeting systems.

Ligand-functionalized liposomal systems often offer more compelling evidence of tissue-specific distribution than traditional formulations, due to the fact that they are logically designed to take advantage of receptor-mediated recognition rather than merely enhancing systemic pharmacokinetics. However, most of the included studies had limited direct mechanistic validation. Nonetheless, since these results might have also been affected by physicochemical characteristics of nanoparticles, circulation kinetics, and non-specific tissue retention, improved cellular uptake or increased tissue accumulation, by themselves, should not be considered definitive proof of successful receptor-mediated targeting [11,48]. Only a small percentage of the included studies examined the mechanistic evidence presented beyond biodistribution, with one study presenting data on receptor competition which directly supported ASGPR-mediated uptake [31]. Therefore, supplementary mechanistic validation approaches such as receptor-blocking or competitive inhibition experiments, as well as quantitative biodistribution assessments on receptor-dependent accumulation, are necessary for robust confirmation of active targeting [11]. Furthermore, since these biological variations may significantly affect the translational potential of ligand-directed liposomal systems, there is need to factor in variabilities in receptor expressions across disease stages, animal models, and human tissues, when interpreting targeting efficiency. Furthermore, direct quantitative comparisons between studies should be interpreted with caution, since oral, intravenous, and intraperitoneal administration routes varied significantly, with likely impact on nanoparticle pharmacokinetics, tissue exposure, and apparent targeting efficiency independent of ligand functionalization. Similarly, variations in NAFLD animal models such as HFD-, MCD-, CCl4, ob/ob-, and ApoE^−/−^-based models, may modify hepatic pathophysiology and receptor expression patterns, thereby impacting nanoparticle biodistribution and restricting cross-study comparisons. Therefore, to determine whether increased hepatic accumulation reflects receptor-mediated targeting rather than non-specific tissue deposition, future studies should integrate quantitative biodistribution analyses with receptor-blocking experiments, cell-specific localization, and pharmacokinetic modelling.

#### 3.4.3. Immune-Evasive Liposomal Strategies

A unique liposomal design approach which is essentially different from traditional receptor-mediated targeting techniques was presented in Tang et al. [26]. This technology was designed to control interactions with the innate immune system, rather than using ligand functionalization to guide liposomes toward cellular receptors. The CD47-functionalized liposomes prolonged circulation and improved liver retention using the CD47–SIRPα signaling axis to decrease Kupffer cell identification and phagocytic uptake. As a result of this immune-evasive architecture, hepatocytes, liver sinusoidal endothelial cells, and other non-phagocytic hepatic cell types accumulated more liposomes. Furthermore, through a decoy-mediated mechanism that reduced macrophage clearance and increased the availability of liposomes for target liver cells, the membrane-engineered variation (MLip) significantly improved intrahepatic delivery.

This platform indirectly enhanced liver delivery by reducing early clearance via the mononuclear phagocyte system (MPS), in contrast to ligand-mediated targeting techniques that depend on receptor-specific identification. Therefore, decreasing macrophage-mediated clearance raised the likelihood of liposome accumulation within hepatic tissue, without necessitating direct receptor-specific interactions. This technique should be viewed as a unique biological delivery strategy since it represents improved hepatic retention, as opposed to traditional receptor-mediated cellular targeting. Moreover, extended systemic circulation may change the composition of the protein corona over time, a situation which may affect cellular interactions and nanoparticle biodistribution without regard to CD47-mediated immune evasion. Therefore, this method offers a different approach to enhancing the distribution of nanocarriers, especially in chronic liver illnesses where the therapeutic efficiency of nanoparticle-based drug delivery systems is often limited by rapid clearance by macrophages.

Despite these benefits, long-term manipulation of the CD47–SIRPα signaling axis may change physiological immune surveillance, impair macrophage homeostasis, and impact nanoparticle clearance after repeated administration. Therefore, immune-evasion strategies need to be carefully considered. Furthermore, since extended circulation may also promote passive accumulation within non-target hepatic compartments, higher hepatic retention alone should not be construed as proof of cell-specific targeting, even when immune-evasive designs may prolong hepatic residence. Thus, little is known about the long-term biological effects and translational importance of these strategies [48,49]. In future research, there is need to address these uncertainties through combination of quantitative biodistribution analysis with cell-type-specific localization, mechanistic validation, and long-term safety evaluations. Such studies are necessary to differentiate between real cellular targeting and immune-evasion-mediated hepatic retention, and also to ascertain if extended nanoparticle persistence will consistently result in greater therapeutic efficacy without impairing physiological immune surveillance. The establishment of this distinction is important for the rational design and successful clinical translation of next-generation liver-targeted liposomal nanocarriers.

### 3.5. Design of Liposomal Nanocarriers, Surface Engineering, and Targeting Techniques in NAFLD/NASH Studies

#### 3.5.1. Conventional Liposomes and Hepatic Passive Delivery

To increase drug solubility, stability, and hepatic exposure, a number of investigations used traditional liposomal formulations without surface functionalization or active targeting ligands, by depending mainly on the inherent benefits of liposomal encapsulation, as listed in Table S2. These studies involved liposomal silybin formulations [14,16], lycopene-loaded nano-liposomes [30], chrysin-loaded nanoliposomes [24], and simvastatin-loaded nanoliposomes [28]. These systems relied on traditional phospholipid/cholesterol vesicles made with well-known fabrication techniques such thin-film dispersion, thin-film hydration, solvent injection, and where necessary, additional size-reduction processes, for example, extrusion [14,16,24,28,30]. The main strategies employed to achieve hepatic exposure in these formulations were systemic biodistribution and improved oral absorption rather than receptor-mediated uptake. The two primary objectives of liposomal silibinin innovation were improved oral bioavailability and increased primary hepatic exposure [16]. Similarly, lycopene- and chrysin-loaded liposomes relied primarily on passive accumulation in the liver [24,30]. These early formulations served as the foundation for the design of more complex liposomal platforms such as surface modification and active targeting techniques, notwithstanding their structural simplicity, relative to later sophisticated targeted systems. However, since pharmacokinetics, protein corona formation, and MPS absorption influence nanoparticle biodistribution, higher hepatic exposure does not always imply liver targeting [11,12].

#### 3.5.2. Engineering Surface Nanocarriers for Oral Delivery

Several studies used polymeric or polysaccharide-based surface modifications to improve intestinal absorption, mucus interaction, and gastrointestinal stability, in order to address the difficulties associated with oral liposomal administration. These surface modifications resulted in chitosan-coated lutein liposomes [19], gum Arabic-coated anthocyanin liposomes [33], spirulina peptide-loaded tosomes [32], and the dextran-coated multilayer oral bilosome DEAE-DEX@SDBS [18]. Surface coatings were used in these systems as protective barriers that improved resistance to gastrointestinal degradation, decreased premature cargo leakage, and increased formulation stability. While DEAE-DEX coating [18] and gum Arabic modification [33] were designed to enhance mucus interaction and oral absorption, chitosan-cosystems [19,32] also showed longer release behavior and better cargo retention.

These approaches mainly enhance oral delivery performance, rather than reflecting receptor-specific hepatic targeting. Polysaccharide coatings may improve mucoadhesion, prolong gastrointestinal residence, and protect cargo against deterioration, depending on surface charge, hydrophilicity, particle size, and coating density. However, in other circumstances, deep mucus penetration may be limited by increased mucus interaction [47]. Furthermore, improved absorption and portal delivery may result in increased hepatic exposure after oral treatment, rather than selective liver targeting. Therefore, until data on biodistribution, receptor-blocking, or mechanistic uptake investigations are presented, the targeting specificity of these surface-engineered oral systems should be carefully assessed, even though they produced considerable formulation benefits [11,12].

#### 3.5.3. Multifunctional and PEGylated Liposomal Platforms

One of the most used surface-engineering techniques in the included studies was PEGylation. The systems that contained PEG were PEGylated vitexin liposomes [20], adipose-targeted T3 liposomes [27], P3-functionalized dasatinib/quercetin liposomes [21], NaA@SC/MAN-LPs [22], GCG-lipo/T3 [23], ROS-responsive liposomes [15], Gal-LNP-RSV [31], and aptamer-functionalized allicin liposomes [25]. PEGylation was used in these formulations to increase colloidal stability, decrease non-specific clearance, extend circulation time, and present a versatile platform for conjugating responsive moieties or targeting ligands. In the mannose [22], glucagon [23], galactose [31], hyaluronic acid [15], aptamers [25], and targeting peptides [21,27] systems, PEGylation was combined with other functions to create multifunctional nanocarriers with potential to integrate stability, targeting, and controlled-release functions.

Despite its advantages, PEGylation is frequently combined with sensitive coatings or targeted ligands, thereby making it difficult to identify its unique contribution. As a result, PEG alone may not always be responsible for improvements in tissue accumulation or biodistribution. Furthermore, a longer circulation does not always provide better target-site delivery, because excessive PEG shielding may reduce cellular uptake, a phenomenon known as the PEG dilemma [50]. Investigations on mechanistic absorption and biodistribution evaluations are still essential for differentiating pharmacokinetic effects from actual targeted activity. Furthermore, direct comparison of biological outcomes is a challenging task, because different studies used different PEG-containing lipid formulations and molecular weights.

#### 3.5.4. Targeting Methods Mediated by Ligands

Some of the included studies used ligand-mediated targeting techniques to improve tissue selectivity and cellular uptake through receptor-specific interactions. Hepatocyte-targeting strategies were used in the generation of galactose-functionalized liposomes [31], which targeted hepatocytes via the asialoglycoprotein receptor (ASGPR), and also in glucagon-modified liposomes [23], which were designed to exploit glucagon receptor-mediated uptake, though this was not directly confirmed by receptor competition experiments. Mannose-modified liposomes were used to study macrophage-directed targeting, Lip/PTM [29] and NaA@SC/MAN-LPs [22], where it was anticipated that mannose receptor-mediated uptake would enhance internalization by hepatic macrophages. Additional receptor-targeting strategies involved peptide-mediated targeting of adipose tissue using PTP- or P3-functionalized liposomes [21,27], hyaluronic acid-coated ROS-responsive liposomes [15], which were designed for CD44-mediated targeting of activated hepatic stellate cells, as supported by HA competition assays. The NAFLD01 aptamer-functionalized allicin liposomes [25] suggested potential CD36-associated targeting, although the absence of receptor competition assays precludes definitive confirmation of CD36-mediated uptake. Enhanced cellular uptake or improved biodistribution alone should not be regarded as conclusive evidence of efficient receptor-mediated targeting, even if ligand-functionalized formulations often offered stronger mechanistic explanation than passive methods [11,47]. For robust confirmation, the excellent methods to use are receptor-blocking studies, mechanistic uptake studies, or quantitative biodistribution analyses showing receptor-dependent accumulation [11].

#### 3.5.5. Advanced Functional Liposomal and Immune-Evasive Systems

A different approach was described by Tang et al. [26]. This involved creating a hybrid liposomal platform that combined collagenase I surface modification with a palmitoylated CD47-derived self-peptide (Pal-self). The technique was developed to avoid Kupffer-cell phagocytosis by modifying the CD47–SIRPα signaling pathway, instead of depending on receptor-mediated absorption. This system is classified as a putative immune-evasive delivery platform rather than a confirmed active targeting system, as it lacked receptor competition or inhibition assays to confirm the specific involvement of the CD47-SIRPα axis in the observed hepatic retention [26]. Thus, hepatic retention was prolonged, with improvement in intrahepatic delivery. This approach marked a shift away from traditional targeting strategies towards more complex multifunctional systems that integrate longer tissue retention, extracellular matrix manipulation, and immune evasion, into a single nanoplatform. Immune-evasion techniques may increase the persistence of nanoparticles in the liver, but their long-term biological effects and translational significance are still not well understood [49,51]. Therefore, there is need for more research to determine whether extended retention consistently results in better therapeutic effects without modifying physiological immune surveillance mechanisms.

### 3.6. Mechanisms of Therapeutic Effects of Liposomal Nanoparticles in NAFLD

**Figure 4:**
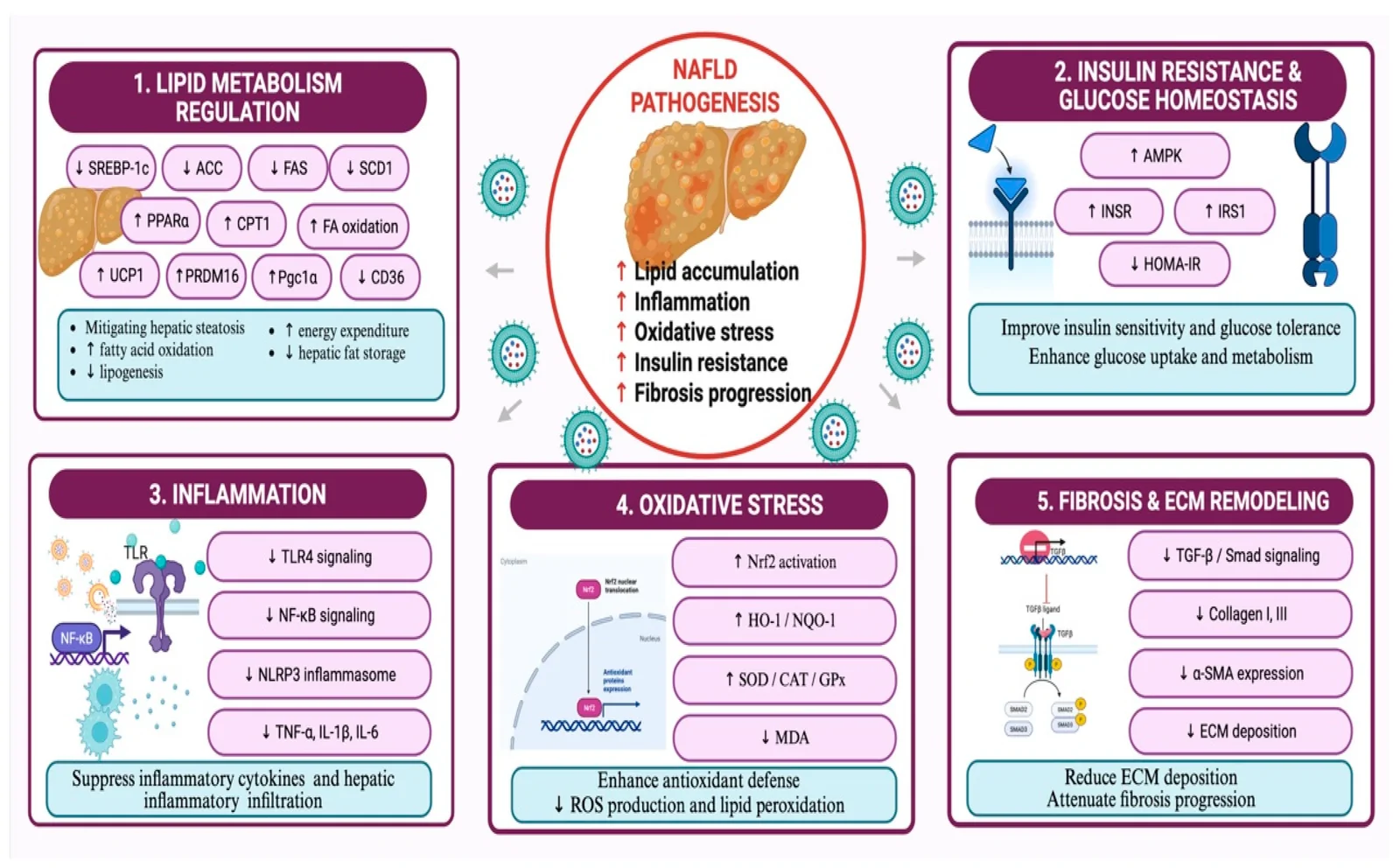
Overview of the therapeutic mechanisms of liposome-based nanotherapies in the management of NAFLD. ↑, increase; ↓, decrease. Created in BioRender. M, S. (2026) https://BioRender.com/myykw09 (accessed on 29 June 2026).

Several shared mechanistic patterns emerged across the various investigations, notwithstanding the substantial disparities among the included studies regarding the administered therapeutic substances (cargo), architecture of the nanostructures, and the targeting strategies employed. Most therapeutic effects were focused on a restricted array of interconnected pathological processes such as disrupted lipid metabolism, insulin resistance, inflammation, oxidative stress, and fibrosis. The AMPK signaling pathway emerged as a primary target among these mechanisms due to its crucial role in managing lipid metabolism, energy homeostasis, and cellular stress responses. Likewise, oxidative stress was a prevalent therapeutic target across various formulations, highlighting its significance as a primary contributor to disease progression. Many advanced liposomal systems simultaneously targeted multiple disease mechanisms, thereby reflecting the multifactorial nature of NAFLD. The molecular mechanisms underlying the therapeutic effects of liposome-based nanomedicines in NAFLD are summarized in Figure 4 and Table S3.

Consequently, the therapeutic effects outlined below were categorized based on the predominant pathological processes frequently addressed in the included studies.

#### 3.6.1. Regulation of Hepatic Lipid Metabolism

**Figure 5:**
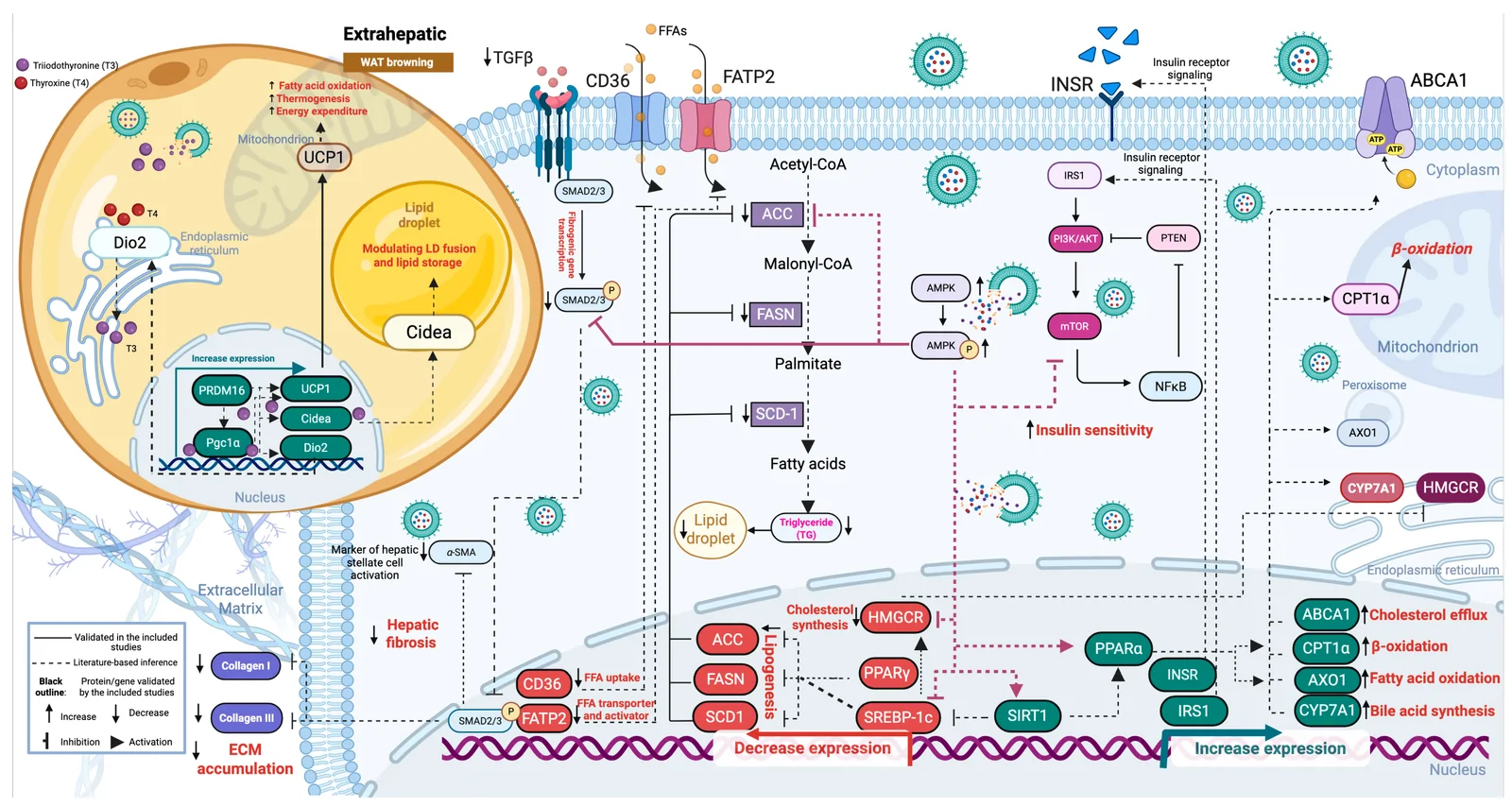
Proposed molecular mechanisms by which liposomal nanoparticles ameliorate NAFLD via metabolic disorders, hepatic steatosis, insulin resistance, and liver fibrosis through coordinated regulation of lipid metabolism, β-oxidation of fatty acids in mitochondria, cholesterol homeostasis, stimulation of adipose tissue browning, and profibrotic signaling. The illustration shows the multi-target therapeutic strategies of liposomal nanoparticles when applied to NAFLD, which correlate with metabolic diseases. These mechanisms involve a simultaneous regulation of hepatic fat absorption, de novo lipogenesis, fatty acid oxidation, insulin signaling, cholesterol metabolism, adipose tissue thermogenesis, and fibrosis formation. In hepatocytes, it reduces fatty acid absorption by decreasing the expression of the membrane transporters CD36 and FATP2. The inhibition of SMAD2/3 phosphorylation diminishes TGF-β-mediated transcription associated with fibrosis, decreases α-SMA expression, and alleviates the accumulation of extracellular matrix components (collagen I and collagen III), thereby ultimately mitigating fibrosis. This illustration demonstrates the suppression of de novo lipogenesis by the downregulation of expressions of ACC, FASN, and SCD1. Collectively, these modifications lead to decreases in triglycerides, lipid droplets, and hepatic steatosis. At the transcriptional level, the suppression of the SREBP-1c and PPARγ pathways downregulates the expression of genes involved in lipid and cholesterol production, and also decreases HMGCR expression. The liposomal formulation stimulates AMPK, which suppresses lipogenesis and promotes metabolic homeostasis. Activation of AMPK suppresses both mTOR and the NF-κB inflammatory pathways, thereby enhancing insulin sensitivity and diminishing inflammation associated with metabolic conditions. Simultaneously, INSR activation activates the IRS1–PI3K/AKT pathway and mitigates the inhibitory influence of PTEN, thereby enhancing efficacy of insulin receptor signaling and improving glucose metabolism. Enhanced oxidative metabolism occurs in response to elevated expressions of PPARα and its downstream targets such as CPT1α and AOX1, leading to higher β-oxidation of fatty acids in both mitochondria and peroxisomes. The concurrent activation of SIRT1 enhances PPARα signaling and ameliorates hepatic metabolic dysfunction. The figure illustrates the management of cholesterol homeostasis through the upregulation of ABCA1, facilitation of cholesterol efflux, and enhancement of CYP7A1 expression which catalyzes the conversion of cholesterol to bile acids. Simultaneously, the inhibition of HMGCR decreases endogenous cholesterol synthesis, thereby enhancing cholesterol homeostasis in the liver. Within white adipose tissue (WAT), the intracellular administration of triiodothyronine (T3) via liposomal nanocarriers (synthesized locally from thyroxine (T4) with the enzyme Dio2) enhances the transcription of thermogenic regulatory genes PRDM16, PGC-1α, UCP1, Cidea, and Dio2. Elevated expression of UCP1 results in enhanced β-oxidation of fatty acids, thermogenesis, and elevated energy expenditure. The Cidea activation concurrently regulates lipid droplet fusion and fat storage, thereby facilitating the conversion of white adipose tissue into brown adipose tissue. Collectively, these coordinated molecular events minimize free fatty acid absorption in the liver, impair de novo lipogenesis and triglyceride accumulation, enhance fatty acid oxidation in mitochondria and peroxisomes, improve insulin sensitivity, elevate cholesterol secretion and bile acid synthesis, stimulate thermogenesis in adipose tissue, and suppress TGF-β/SMAD-dependent fibrosis. Consequently, hepatic steatosis, inflammation, insulin resistance, extracellular matrix deposition, and the progression of fibrosis, are reduced. Created in BioRender. M, S. (2026) https://BioRender.com/tr4szr8 (accessed on 29 June 2026).

The fundamental pathogenic characteristic of NAFLD is the accumulation of fat within the hepatocytes, resulting from a complicated imbalance among de novo lipogenesis, fatty acid absorption, fatty acid oxidation, and cholesterol metabolism. As shown in Figure 5, the studies included in this review demonstrated that liposome-based nanosystems mitigated hepatic fatty degeneration by modulating several interrelated metabolic pathways, rather than focusing on just one pathway. The inhibition of de novo lipogenesis was among the most employed therapeutic approaches in the included studies. Platensimycin-loaded liposomes showed efficacy in diminishing triglyceride accumulation in the liver by inhibiting fatty acid synthase (FASN), the principal enzyme involved in fatty acid biosynthesis. This was accompanied by reductions in the expressions of SREBP-1c, ACC, and SCD1, alongside upregulations of the expressions of PPARα and ACOX1, indicating inhibition of lipid biosynthesis and enhancement of fatty acid oxidation [29]. Similarly, Aptamer-functionalized Allicin-loaded liposomes downregulated the gene expressions of ACC, FAS, SCD-1, SREBP1-C, and PPARγ, and lowered CD36 while restricting hepatocyte fatty acid uptake [25].

Several studies revealed that activation of the AMPK signal pathway is a crucial switch for restoration of metabolic balance to the liver. Liposome silybin enhanced glucose and lipid metabolism in the T2DM-NAFLD model through activation of AMPK and inhibition of the TGF-β1/Smad pathway [14]. Similarly, dual-targeting sodium acetate-loaded liposomes triggered hepatic AMPK signaling, reduced the expressions of Acc1, Fasn, Scd1, and Srebf1, and increased CPT1α, resulting in enhanced fatty acid oxidation and decreased hepatic fat storage [22]. Nanoliposomes loaded with spirulina peptide provided the most complete evidence of AMPK-mediated metabolic regulation. These formulations increased AMPK phosphorylation, and upregulated PPARα, CPT-1, AOX1, SIRT1, ABCA1, and CYP7A1, while decreasing SREBP-1c, FAS, and ACCα. These effects led to a metabolic shift in the liver towards increased lipid intake and decreased lipid storage [32].

In addition to directly impacting hepatic fat production, certain liposomal systems showed capacity to decrease steatosis via targeting metabolic tissues outside the liver. The T3-loaded liposomes converted white adipose tissue into thermogenic tissue by increasing the expressions of UCP1, Dio2, Pgc1α, Cidea, and PRDM16. This led to increased energy expenditure, reduced inflammation in adipose tissue, and reduced fat accumulation in the liver [27]. Glucagon-modified liposomes loaded with T3 mitigated obesity-related fatty liver by targeting the liver, increasing browning in adipose tissue, and simultaneously improving glucose tolerance and insulin sensitivity [23]. These findings suggest that the liver-adipose axis is an indirect but effective therapeutic target for NAFLD. Likewise, adipose tissue-targeted liposomal serotherapy demonstrated that extrahepatic targeting approaches may significantly diminish hepatic steatosis. White adipose tissue-targeted liposomes containing dasatinib and quercetin specifically targeted senescent cells in adipose tissue, thereby arresting WAT lipolysis and decreasing the release of free fatty acids (FFAs) into the circulatory system. The inhibition of lipolysis in adipose tissue significantly diminished hepatic steatosis and decreased hepatocyte senescence, as the excessive release of fatty acids from adipose tissue is a primary contributor to fat accumulation in the liver [21].

Some liposomal formulations exhibited potential to improve lipid metabolism via broader metabolic pathways. Silybinin liposomes lowered the expression levels of CD36, FATP2, FAS, and SREBP-1 while enhancing insulin sensitivity and decreasing lipid accumulation hepatic tissue [16]. Oxidative stress-responsive liposomes containing LY294002 and oridonin upregulated the expressions of IRS1 and INSR while decreasing oxidative stress, inflammation, and hepatic fat accumulation [15]. Blueberry anthocyanin-loaded liver-targeted nanoparticles (ACNs-LNP) minimized intracellular lipid droplet accumulation, improved blood lipid profile, and modulated intestinal microbiota disruption [33], Lutein liposomes reduced liver triglyceride and cholesterol levels and weakened liposomes via antioxidant stress effects [19].

In addition to pathway-oriented formulations, PEGylated vitexin-loaded liposomes significantly reduced cirrhosis-related symptoms, and improved liver histology and liver function metrics in a NASH/NAFLD-associated animal model [20]. These therapeutic benefits were primarily attributed to higher bioavailability of vitexin, extended drug release, and enhanced pharmacokinetic behavior as a result of its encapsulation within PEGylated liposomes. Although no specific molecular pathways were experimentally validated in the study, the PEGylated vitexin-loaded formulation was effective in alleviating disease severity, with higher therapeutic efficacy than free vitexin and non-PEGylated liposomes. This emphasizes the critical importance of optimizing nanocarrier design in improving therapeutic outcomes.

Apart from enhancing bioavailability, active hepatocyte-targeting strategies further improved therapeutic efficacy. Galactose (Gal)-modified lipid nanoparticles (Gal-LNPs) were employed to augment hepatic targeting through asialoglycoprotein receptor-mediated endocytosis, leading to enhanced hepatic uptake and enhanced efficiency of intracellular delivery. This specific regimen diminished liver fat accumulation and enhanced liver disease markers more effectively than free resveratrol. The enhanced therapeutic outcomes were associated with the targeted activation of intracellular mechanisms, which restored metabolic and redox equilibrium, thereby underscoring the significance of receptor-mediated targeting strategies in improving the efficacy of liposome-based nanotherapies for hepatic steatosis [31].

Overall, the studies reviewed here have demonstrated that liposome-based nanoparticles are a viable approach to alleviating hepatic steatosis by regulating adipose-liver metabolic crosstalk, lowering fatty acid uptake, improving β-oxidation, regulating cholesterol metabolism, and inhibiting de novo lipogenesis. Liposomal nanomedicines are capable of simultaneously targeting numerous primary determinants of NAFLD development, as evidenced by their multifactorial effects. Consequently, they provide superior treatment efficacy, relative to conventional free-drug formulations.

#### 3.6.2. Reduction of Insulin Resistance and Improvement of Glucose Homeostasis

Insulin resistance is an important factor in the progression of NAFLD, since it leads to increase de novo lipogenesis, reduced utilization of glucose, and aggravation of chronic metabolic conditions. Consequently, numerous liposome-based nanoparticles have been developed to specifically enhance insulin sensitivity and restore glucose hemostasis as components of therapeutic approaches to NAFLD.

In one of the studies included in this review, liposomal silybin produced significant efficacy in enhancing insulin resistance in T2DM-related NAFLD model. The activation of the AMPK signaling system enhanced glucose utilization, augmented hepatocyte glucose uptake, decreased fasting blood glucose levels, and produced good outcomes in oral glucose tolerance test (OGTT) and insulin tolerance test (IPITT). These metabolic enhancements were accompanied by significant decreases in hepatic fibrosis, thereby highlighting the crucial function of AMPK activation in restoration of metabolic balance [14]. In a comparable manner, silibinin-loaded liposomes enhanced insulin sensitivity by modulating several genes linked to insulin signaling and glucose metabolism. Transcriptomic studies revealed modification of intrahepatic insulin resistance genes such as Socs3, Slc2a2, Ptprf, Cpt1a, CD36, and IRS1. These changes were associated with reduced fasting insulin levels and a lower HOMA-IR index, signifying a considerable enhancement of systemic insulin sensitivity [16].

One of the most in-depth studies involved ROS-responsive liposomes containing both LY294002 and oridonin. These nanoparticles restored several components of the insulin signaling system, i.e., IRS1, INSR, SOCS3, and PTEN while reducing oxidative stress and inflammatory signaling. By correcting multiple associated metabolic abnormalities, this formulation reduced insulin resistance and hepatic fat accumulation, and slowed down disease development [15]. Dual-targeted sodium acetate-loaded liposomes showed multifaceted metabolic advantages. Hepatic AMPK activation improved glucose tolerance and diminished fasting glucose-related metabolic abnormalities while decreasing lipogenesis and improving fatty acid oxidation. These data indicate a close interconnection among therapy outcomes for NAFLD, glucose homeostasis improvement, and lipid regulation [22].

Certain liposomal systems were linked to enhancement of insulin sensitivity via indirect mechanisms by ameliorating obesity-linked metabolic dysfunctions. For instance, it was demonstrated that adipose tissue-targeted T3-loaded liposomes stimulated the browning of white adipose tissue by upregulating the expressions of the thermogenes UCP1, Dio2, Pgc1α, Cidea, and Prdm16. These upregulations resulted in increased energy expenditure, diminished adipose inflammation, and raised adiponectin secretion, thereby enhancing insulin sensitivity and reducing hepatic lipid accumulation [27]. Similarly, glucagon-modified T3 liposomes enhanced glucose tolerance and insulin sensitivity while decreasing obesity-related hepatic steatosis and dyslipidemia [23]. Lycopene-loaded nanoliposomes exhibited significant anti-diabetic properties and enhanced insulin sensitivity. The lycopene nanoliposome treatment resulted in lowered levels of fasting glucose, fasting insulin, and HOMA-IR, along with increased adiponectin and decreased leptin levels. These results were linked to the stimulation of AMPK signaling and the normalization of autophagy, indicating that the manipulation of energy-sensing pathways is crucial for enhancing metabolic function [30]. Similarly, spirulina peptide-loaded nanoliposomes reduced levels of blood glucose and HOMA-IR through AMPK activation and fatty acid β-oxidation [32].

Evidence indicates that liposome-based nanoparticles enhanced insulin resistance and glucose homeostasis via various integrated mechanisms: activation of AMPK signaling, restoration of insulin signaling pathways, increases in adiponectin secretion, reductions in obesity-related inflammation, and decreases in oxidative stress. Liposomal nanomedicines markedly broke the vicious cycle of insulin resistance, hepatic steatosis, and NAFLD progression by targeting these inter-linked metabolic disorders.

#### 3.6.3. Anti-Inflammatory Effects

**Figure 6:**
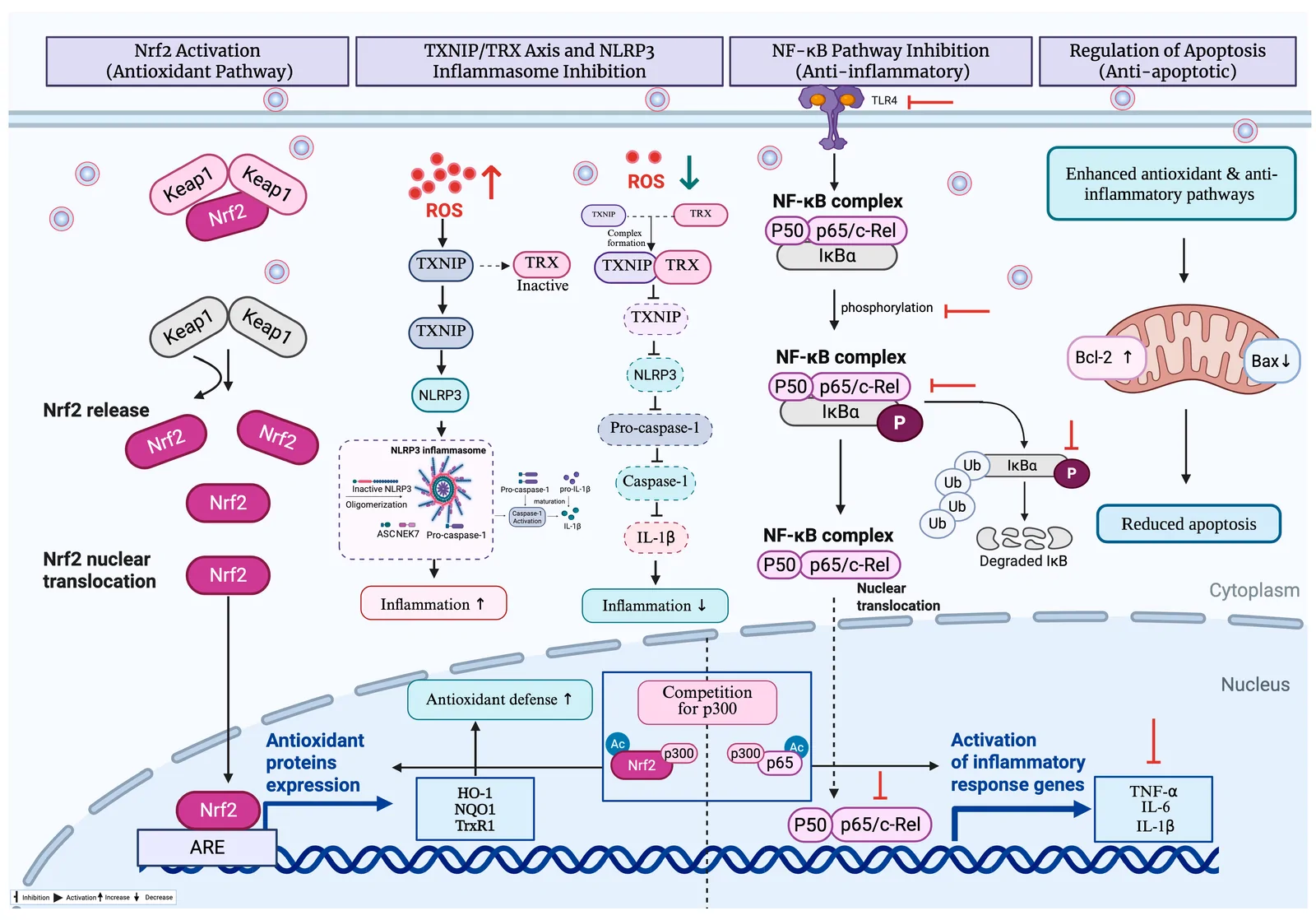
Proposed molecular mechanisms for hepatoprotective effects involve the coordinated activation of antioxidant pathways and the inhibition of inflammatory and apoptotic signals. The graph highlights four interrelated mechanisms by which treatment minimizes oxidative stress, inflammation, and apoptosis: 1. Activation of the Nrf2 signaling pathway (antioxidant pathway): Under normal conditions, the transcription factor Nrf2 is sequestered in the cytoplasm by the Keap1 protein. Upon activation, Nrf2 dissociates from Keap1 and translocates to the nucleus, where it induces the transcription of antioxidant and protective genes (HO-1, NQO1, and TrxR1). The heightened expressions of these proteins enhance the cellular antioxidant defense mechanism, thereby mitigating oxidative stress. 2. Regulation of the TXNIP/TRX axis and inhibition of the NLRP3 inflammasome complex: Oxidative stress induces the dissociation of TXNIP from TRX, thereby enabling TXNIP to associate with and activate the NLRP3 inflammasome complex which subsequently activates caspase-1, facilitates IL-1β maturation, and enhances the inflammatory response. Liposome-based nanotherapy diminishes intracellular ROS levels, stabilizes the TXNIP–TRX complex, inhibits TXNIP-mediated NLRP3 activation, suppresses caspase-1 activation, and prevents IL-1β maturation, resulting in lowering of inflammasome-dependent inflammation. 3. Inhibition of the NF-κB pathway (anti-inflammatory pathway): In pathological conditions, the activation of inflammatory signaling results in the phosphorylation of p65 and the phosphorylation and degradation of the protein IκBα, a process facilitated by the ubiquitin system. This process permits the translocation of the NF-κB complex to the nucleus, thereby stimulating the transcription of inflammatory cytokines such as TNF-α, IL-6, and IL-1β. Liposome-based nanotherapy suppresses the phosphorylation of p65 and IκBα, and blocks the degradation of IκBα, thereby obstructing the translocation of NF-κB to the nucleus and suppressing the expressions of inflammatory genes. Simultaneously, activated Nrf2 competes with NF-κB for the transcriptional cofactor p300, resulting in reduction of transcriptional activity of NF-κB and subsequently enhancing the transcriptions of antioxidant genes. 4. The therapy modulates apoptosis through the anti-apoptotic pathway by concurrently amplifying antioxidant and anti-inflammatory signals. This regulation affects mitochondria-associated apoptotic proteins by upregulating the expression of the anti-apoptogenic protein Bcl-2 and downregulating the expression of the pro-apoptotic protein Bax, thereby decreasing the incidence of apoptosis. Overall, the activation of the Nrf2/ARE antioxidant pathway, inhibition of the ROS–TXNIP/TRX–NLRP3 inflammasome axis, suppression of the TLR4/NF-κB pathway, and modulation of the Bcl-2/Bax apoptosis pathway enhance antioxidant defenses, result in decreased production of inflammatory cytokines, and protect cells from oxidative stress and apoptotic damage. Created in BioRender. M, S. (2026) https://BioRender.com/el26r8d (accessed on 29 June 2026).

Chronic low-grade inflammation is a crucial element in the pathogenesis of NAFLD because it drives the progression from hepatic steatosis to steatohepatitis, which subsequently leads to fibrosis. Continuous stimulation of inflammatory pathways leads to increased generation of pro-inflammatory cytokines, enhances immune cell infiltration, causes damage to hepatocytes, and induces extracellular matrix remodeling. Therefore, various liposome-dependent nanosystems have been designed to reduce inflammatory signaling and ameliorate NAFLD-related metabolic abnormalities (Figure 6). The co-delivery method of berberine and curcumin demonstrated one of the most extensive anti-inflammatory mechanisms among the studies included in this review. The DEAE-DEX-coated bilosomes increased Nrf2 signaling while suppressing NF-κB activation through reductions in the phosphorylation of p65 and IκBα. Furthermore, the DEAE-DEX-coated bilosomes inhibited the activation of the NLRP3 inflammasome by downregulating the expressions of NLRP3, Caspase-1, and IL-1β. The concurrent control of oxidative stress and inflammation resulted in hepatic inflammation and overall amelioration of NAFLD pathologies [18]. The NF-κB signaling pathway was a frequently targeted inflammatory route in the articles reviewed. Silibinin-loaded liposomes reduced hepatic inflammation by inhibiting NF-κB-associated genes and inflammation-associated mediators. This was accompanied by significant reductions in IL-6, IL-1β, and TNF-α levels, as well as macrophage infiltration [16]. Liposomes containing LY294002 and/or oridonin lowered NF-κB activation, reduced levels of TNF-α and IL-6 while alleviating hepatic inflammation, insulin resistance, and oxidative stress [15].

Some of the included studies were specifically focused on innate immunological signaling pathways. Nanoliposomes encapsulated chrysin showed efficacy in obstructing the TLR4 signaling pathway, thereby blocking the downstream activation of p38 and NF-κB. Consequently, the expression levels of TNF-α and IL-6 were reduced. These liposomes also improved macrophage infiltration and neutrophil accumulation; decreased hepatic expression of myeloperoxidase (MPO, a crucial enzyme in the enzymatic production of ROS); and reduced oxidative stress levels. These results highlight the extensive efficacy of nanoliposomes in mitigating NASH-associated inflammation [24]. Many studies addressed inflammation mediated by Kupffer cells which are crucial immune cells in the development of NAFLD. Sodium cholate and mannose-modified sodium acetate-loaded liposomes were designed and used to target hepatocytes and Kupffer cells. As a result, there were significant decreases in the expressions of IL-1β, IL-6, and TNF-α, while the expression of anti-inflammatory IL-4 was markedly increased. Furthermore, macrophage infiltration was significantly reduced, as was NF-kB activation, thereby indicating the importance of focusing on Kupffer cells in controlling hepatic inflammation [22]. One study presented a distinct and innovative methodology for the immune-evasive hybrid liposomal platform. This platform diminished macrophage over-activation through interaction with SIRPα receptors on Kupffer cells using CD47-derived self-peptide. The treatment significantly reduced hepatic macrophage infiltration, IL-1β generation, IL-6 secretion, ROS formation, and inflammatory tissue remodeling. The study demonstrated that immune evasion provided strong anti-inflammatory advantages for NAFLD nanotherapy [26].

Alternative formulations also exhibited anti-inflammatory advantages indirectly by mitigating oxidative stress and metabolic abnormalities. Lycopene-loaded nanoliposomes inhibited TNF-α, IL-6, IL-1β, and COX-2, while promoting autophagy and reducing Hedgehog signaling [30]. Nanoliposomes containing spirulina peptides reduced levels of TNF-α, IL-1β, and IL-6 while improving antioxidant systems and hepatic lipid metabolism [32]. Similarly, simvastatin-loaded nanoliposomes reduced the secretions of IL-1α, IL-1β, IL-6, and TNF-α, and also decreased ROS production and collagen I deposition in an NAFLD model of fibrosis [28]. Overall, the experimental evidence indicated that liposome-based nanoparticles effectively inhibited hepatic inflammation through various complementary mechanisms. These mechanisms comprised inhibition of NF-κB signaling, suppression of TLR4-mediated innate immune activation, regulation of NLRP3 inflammasome activity, modulation of Kupffer cell function, reduction of inflammatory cytokine production, and mitigation of oxidative stress. Thus, liposomal nanomedicines targeting inflammatory pathways and immune cells involved in the NAFLD have the potential to interrupt the progression of the NAFLD inflammatory cascade towards NASH and fibrosis.

#### 3.6.4. Antioxidant Effects and Redox Homeostasis

Oxidative stress is a major contributor to the pathogenesis of NAFLD through the association among hepatic steatosis, inflammation, insulin resistance, mitochondrial dysfunction and fibrosis. The overproduction of ROS causes lipid peroxidation, and cellular damage promotes the secretion of inflammatory cytokines while stimulating the pathways that result in fibrosis. Therefore, restoration of redox equilibrium has become a primary therapeutic target for liposome-based nanosystems, as depicted in Figure 6. The co-delivery mechanism of berberine and curcumin produced one of the most mechanically efficient antioxidant systems. The DEAE-DEX-coated bilosomes stimulated facilitated the translocation of Nrf2 into the nucleus and the production of its antioxidant enzymes, i.e., NQO-1, HO-1, and TrxR-1. Concurrently, these nanoparticles facilitated the TXNIP–TRX reaction and inhibited the TXNIP–NLRP3 pathway, resulting in diminished oxidative stress and inflammatory activation. These effects were associated with reduced MDA levels and enhanced antioxidant enzyme activity in NAFLD mice models [18].

Other investigations revealed a direct capacity of some liposome formulations to significantly eliminate ROS and increase endogenous antioxidant defenses. Spirulina peptide-loaded nanoliposomes markedly decreased various oxidative stress markers such as protein carbonyls (PCO), advanced oxidation protein products (AOPPs), advanced glycation end products (AGEs), and MDA. These improvements were associated with elevations in the hepatic activities of GPx and SOD, demonstrating improved antioxidant capacity [32]. In addition, chrysin-loaded nanoliposomes decreased ROS production and MPO expression, and also suppressed inflammatory cell infiltration and hepatic fibrosis [24]. The therapeutic effects of lutein-loaded liposomes were mostly based on antioxidant mechanisms. These particles decreased lipid peroxidation, enhanced activity of antioxidant enzymes such as CAT, SOD and GSH-Px; reduced intracellular ROS generation, and enhanced mitochondrial membrane potential. In HFD-induced NAFLD animal models, these effects were evident in decreases in fat vacuoles and fat accumulation [19]. Similarly, lycopene-loaded nanoliposomes showed antioxidant effects by decreasing MDA levels and increasing total antioxidant capacity (TAC). More importantly, the lycopene-loaded nanoliposome therapy restored oxidative equilibrium through the activation of AMPK-associated pathways and inhibition of inflammatory mediators, thereby improving liver tissue architecture and metabolic parameters [30].

The creation of ROS-responsive liposomes is considered an interesting and novel method for managing oxidative stress. The RLLs system contained a thioketal linker that directly scavenged excessive ROS while simultaneously facilitating the controlled release of LY294002 and oridonin under conditions of elevated oxidative stress. These particles also regulated the activities of antioxidant enzymes such as SOD, catalase, and GSH-Px; diminished oxidative damage, and augmented CYP3A11 activity. This dual mechanism facilitated the eradication of oxidative stress and the targeted drug delivery to the damaged hepatic tissues [15]. Blueberry anthocyanin-loaded liposomal nanoparticles demonstrated additional antioxidant advantages by reducing ROS accumulation in cells to a certain threshold, maintaining antioxidant activity during gastrointestinal digestion, and mitigating hepatic oxidative damage in animals administered a high-fat diet. These effects were associated with enhanced lipid metabolism and gut microbiota composition, indicating broader systemic impacts on the regulation of oxidative stress [33]. The immune-evasive hybrid liposomal platform displayed ROS-scavenging effects in activated macrophages. The treatment decreased intracellular ROS production and simultaneously inhibited the development of inflammatory cytokines and fibrosis, which indicates the close association between oxidative stress and chronic hepatic inflammation [26]. Overall, these data suggest that liposome-based nanomedicines have a significant therapeutic effect on oxidative stress. Most of the antioxidant-focused liposomal systems reduced ROS levels and improved cellular defenses against oxidative stress, regardless of cargo composition or formulation design. The findings suggest that redox modulation may serve as an important mechanistic foundation for explaining the numerous beneficial therapeutic effects observed in the various liposomal treatments for NAFLD.

#### 3.6.5. Anti-Fibrotic Mechanisms

Hepatic fibrosis is a pivotal stage in the advancement of NAFLD, and it serves as a significant predictor of liver disease-associated morbidity and mortality. Chronic inflammation, oxidative stress, hepatic cell damage, and the activation of hepatic stellate cells (HSCs) lead to enhanced extracellular matrix deposition and collagen accumulation. Consequently, various liposome-based nanotherapeutic strategies were formulated to inhibit the pathways associated with fibrosis and to obstruct the pathological remodeling of hepatic tissue (Figure 5).

In one of the studies under consideration, liposome-encapsulated silybin demonstrated antifibrotic activity by modulating the AMPK/TGF-β1/Smad pathway in a T2DM-NAFLD rat model. The activation of AMPK was associated with the inhibition of TGF-β1 expression and reductions in the phosphorylation of Smad2 and Smad3. At the same time, there were decreases in the expressions of collagen I, collagen III, and α-SMA, all of which are critical markers of hepatic astrocyte activation and extracellular matrix production. Histological analyses revealed a decrease in severity of fibrosis, and enhancement in liver tissue architecture [14]. In the same manner, the oxidative stress-responsive liposomal system loaded with both LY294002 and Oridonin, demonstrated an anti-fibrotic effect by reducing the degree of fibrotic lesions through the simultaneous targeting of multiple pathological routes such as oxidative stress, inflammation, insulin resistance, and fibrosis. Treatment with the oxidative stress-responsive liposomal system suppressed the TGF-β pathway, decreased collagen deposition, lowered the expression of collagen IV (COL4), and enhanced fibrosis scores in CCl4-induced NAFLD murine models. These effects underscore the significance of multi-faceted approaches in managing hepatic fibrosis.

One of the most innovative strategies for balancing immune evasion with target cells was developed by Tang et al. [26] by designing a tunable mixed system (Mlip) that combined conventional liposomes (Lip) and self-peptide-modified liposomes (Slip) at variable ratios. This mixed system inhibited phagocytosis in a dose-dependent manner, with higher SLip content resulting in lower Kupffer cell uptake. SLip effectively avoided Kupffer cell clearance while maintaining tight attachment to hepatic membranes, thereby separating immune escape from cellular uptake. This allowed for prolonged circulation and improved drug retention in the liver. To evaluate its therapeutic efficacy, silibinin was encapsulated in MLip, and ECM remodeling was induced through surface absorption of collagenase I (C1). The results showed significantly greater anti-fibrotic activity with C1-MLip than with C1-SLip, with the fibrotic area in the C1-Mlip group approaching that of the LFD control. Furthermore, the study found that F4/80, a macrophage activation marker, and collagen I, the major structural component of the fibrotic ECM, were markedly upregulated in the PBS group, but markedly downregulated in the C1-MLip/silibinin group. This demonstrates the potent anti-inflammatory and antifibrotic effects of the treatment, which were most likely mediated by inhibition of macrophage induction, as well as HSC-driven ECM generation [26].

Simvastatin-loaded nanoliposomes prevented the advancement of liver fibrosis. The activation of the KLF2–NO axis enhanced nitric oxide (NO) production, decreased oxidative stress, and suppressed the release of inflammatory cytokines, thereby reducing collagen I expression and extracellular matrix accumulation. At reduced concentrations, the liposomal formulation of simvastatin produced higher antifibrotic efficacy than the free drug, thereby highlighting the therapeutic advantages of nanosystems [28]. Chrysin-loaded nanoliposomes produced antifibrotic activity by inhibiting the TLR4-mediated inflammatory signaling pathway. Inhibition of TLR4, p38, and NF-κB reduced the synthesis of inflammatory cytokines and decreased inflammatory cell infiltration, oxidative stress, and collagen deposition. The treatment markedly diminished fibrosis scores and a-SMA expression in the MCD diet-induced NASH model [24]. Although silibinin-loaded liposomes were not intended to be anti-fibrosis therapies, secondary antifibrosis benefits were demonstrated, as were evident in reduction of hepatic collagen deposition in Masson staining, as well as improved insulin resistance, inflammation, and hepatic steatosis [16].

Collectively, the included studies indicate that liposome-based nanoparticles may mitigate fibrosis via various mechanisms such as inhibition of the TGF-β/Samad pathway, suppression of hepatic astrocyte activation, reduction of collagen synthesis, modulation of inflammatory pathways and oxidative stress, activation of the KLF2–NO axis, and direct remodeling of the extracellular matrix. These effects are considered significant since fibrosis is the primary determinant of long-term clinical outcomes in NAFLD patients [52]. Therefore, multi-target liposomal systems constitute a superior alternative to conventional single-target therapies.

## 4. Translational Barriers and Evidence Gaps

Despite the encouraging preclinical findings, the therapeutic superiority of advanced liposomal formulations over conventional liposomes remains inconclusive, primarily due to substantial methodological heterogeneity across the 19 included studies. This variability encompassed diverse animal models (HFD, MCD, CCl_4_, ob/ob, ApoE^−^/^−^), disease induction protocols, treatment durations (ranging from single-dose acute administration to 12-week chronic regimens), dosing strategies, administration routes, and outcome assessment methods. Critically, several studies relied heavily on cell line models (e.g., HepG2, LX-2) without corresponding in vivo validation for key mechanistic pathways, limiting the translational relevance of their findings. Moreover, mechanistic evidence was inconsistent: some studies provided comprehensive molecular analyses, while others relied solely on biochemical or histological endpoints without establishing causal relationships. The lack of direct comparative studies further confounded the interpretation of the true advantages of the advanced systems. Major biological hurdles persist, including rapid clearance by the mononuclear phagocyte system, protein corona formation, and restricted penetration through fibrotic ECM in advanced disease. Manufacturability and regulatory challenges are equally pressing; thin-film hydration was predominant in the included studies, while scalable technologies such as microfluidic assembly [31] and high-pressure homogenization [32] were rarely used. A further major limitation of the current evidence base is the inconsistent and often incomplete reporting of quantitative nanoparticle characterization data across primary studies. Critical parameters such as particle size, polydispersity index, zeta potential, encapsulation efficiency, drug loading, and release kinetics were frequently underreported or entirely omitted in several studies [15,27,28]. This lack of standardized characterization not only impeded direct cross-study comparisons but also limited the ability to establish reliable structure–activity relationships and to identify formulation attributes predictive of therapeutic efficacy. Accordingly, we emphasize the need for comprehensive and standardized reporting of nanoparticle properties in future preclinical studies to enhance data comparability, reproducibility, and translational potential. Critically, all evidence presented was preclinical: no published data on clinical trials were presented. Pharmacokinetics, biodistribution, immunogenicity, complement activation, chronic toxicity, repeated-dose effects, and off-target accumulation in humans were entirely unassessed. The animal models used do not completely reflect the complexity and heterogeneity of human NAFLD. Additionally, it is possible that publication bias led to overestimation of efficacy due to underreporting of unsuccessful formulations or negative results.

### 4.1. Manufacturing Techniques and Critical Quality Attributes

Across the studies included, the methods of preparation varied significantly, with thin film hydration being the technique used in 11 out of 19 studies. Other methods included ether injection [30], microfluidic mixing [31], high-pressure homogenization [32], dehydration-rehydration [29], and freeze-drying [28]. This diversity has direct im-plications for critical quality attributes (CQAs), including batch-to-batch reproducibility, polydispersity index (PDI), and entrapment efficiency (EE). Although thin-film hydration is versatile, it has limited reproducibility between batches due to its sensitivity to processing parameters. This is evident in the highly variable PDI values (ranging from <0.15 to >0.5) and broad EE ranges (38–95%) reported in various studies [14,16,18,19,20,21,22,23,24,25,26]. Conversely, microfluidic mixing [31] yielded an excellent PDI (<0.15) and high EE (>85%), but it was utilized in only one study, indicating a significant gap in its adoption for scalable manufacturing. High-pressure homogenization [32] and ether injection [30] were infrequently utilized, and neither method underwent a systematic assessment for reproducibility. Dehydration-rehydration [29] and freeze-drying [28] were used in separate studies, each with distinct scalability challenges. There are considerable translational worries regarding laboratory-scale thin-film hydration, given that processes that cannot be scaled up and that lack reproducibility are probably unable to satisfy regulatory requirements. Cross-study comparisons and the identification of predictive formulation attributes are further hindered by the lack of standardized protocols and systematic evaluations of critical process parameters. To sum up, non-scalable techniques with inadequately managed CQAs are predominant in the current evidence base. To enable clinical translation, future studies should focus on scalable manufacturing methods that comply with GMP standards and include thorough characterization of CQA, with data on reproducibility across batches.

### 4.2. Liposomal Formulations’ Safety and Biocompatibility’

The 19 included studies have conflicting safety and biocompatibility findings. Regarding extrahepatic organ histology and systemic safety, only four studies [18,20,22,27] provide hard data. Chen et al. [18] reported minimal cytotoxicity against LO2 cells (<10% inhibition); Farooq et al. [20] showed improved histopathology in liver, kidney, and spleen with PEGylated vitexin liposomes; Hou et al. [22] showed good tolerability (>85% cell viability); and Chen et al. [27] reported no toxicity to the heart or skeletal muscle. The remaining fifteen studies merely provided qualitative safety claims rather than systematic histological evaluations of non-target organs. While some research [14,20,24,26,27,29,30,33] did not disclose cytotoxicity data and instead relied on in vivo observations, the majority of studies [15,16,18,19,22,25,28,31,32] showed favorable profiles with cell viability >80% at therapeutic doses. It is important to note that none of the 19 trials evaluated immunogenicity, long-term toxicity, accelerated blood clearance (ABC), repeated-dose pharmacokinetics, or anti-PEG antibodies. This is particularly worrying as PEGylated formulations made up a significant portion of the studies included [15,20,21,22,23,25,26,27,31], and the ABC phenomenon is a known translational barrier [53,54]. To sum up, while the evidence suggests that liposomal formulations are usually well tolerated, essential safety information is consistently absent. Reliable determination of translational potential requires future studies to focus on comprehensive toxicological investigation, which must encompass histopathological analysis of all key organs, pharmacokinetic studies involving repeated dosing, and evaluation of anti-PEG antibodies and the ABC phenomenon.

### 4.3. Model Heterogeneity and Clinical Translatability

The included studies exhibited considerable heterogeneity in animal models, with the HFD model predominating 13 of 19 studies [14,16,18,19,21,22,25,26,27,30,31,32,33], while the MCD diet [24], CCl_4_-induced fibrosis models [15,20,29], and the genetic ob/ob model [23] were rarely used. Importantly, formulations effective in HFD-induced steatosis—predominantly metabolic improvements—did not consistently demonstrate comparable efficacy in advanced disease models. For instance, chrysin-loaded nanoliposomes showed potent anti-inflammatory and anti-fibrotic effects in the MCD model [24], whereas formulations tested exclusively in HFD models exhibited little or no evidence of fibrosis resolution. Additionally, while the genetic ob/ob model [23] depicts extreme obesity, it does not include the critical dietary environmental factor that contributes to the development of NAFLD in humans. Critical questions about the generalizability of existing preclinical research to the diverse range of human NAFLD/NASH are raised by this model-dependent heterogeneity. Therapeutic benefits shown in HFD models may overstate clinical effectiveness in individuals with advanced NASH because these models lack the strong inflammatory and fibrotic elements typical of progressing illness. To generate solid proof of wide translatability, future research should assess liposomal formulations across complementary models that replicate the whole pathophysiological range of NAFLD, including fibrosis and hepatocellular damage.

## 5. Future Perspectives and Translational Priorities

Research must move beyond incremental optimization toward clinically translatable platforms that integrate efficacy, manufacturability, safety, and regulatory feasibility from the earliest stages. A “Design for Translation” approach is essential for balancing increasing system complexity with practical manufacturing and regulatory requirements. Studies should systematically integrate physicochemical characterization (size, surface charge, encapsulation efficiency, and release kinetics) with nano-biointeractions, pharmacokinetics, biodistribution, cellular uptake, protein corona formation, and long-term therapeutic outcomes, in order to identify predictive formulation attributes. Standardized manufacturing protocols employing scalable technologies such as microfluidic assembly and continuous manufacturing, are urgently needed. Computational modeling offers opportunities to accelerate optimization by predicting relationships between composition, properties, and performance while reducing cost and development time. Given the marked metabolic, inflammatory, and fibrotic heterogeneity of NAFLDS, future strategies must shift toward precision nanomedicine by tailoring liposomal formulations to disease stage and patient phenotype, rather than adopting a one-size-fits-all approach.

## 6. Conclusions

Liposomal nanocarriers hold considerable therapeutic promise for NAFLD through coordinated multi-pathway regulation, with focus on the AMPK, NF-κB, Nrf2, TGF-β/Smad, TLR4, and PI3K/AKT signaling routes. This strategy will concurrently reduce steatosis, insulin resistance, oxidative stress, inflammation, and fibrosis. However, clinical translation is significantly constrained by biological barriers (phagocytic clearance, protein corona, and ECM fibrosis); manufacturing challenges (scale-up, batch reproducibility, and limited long-term stability), and complete absence of clinical safety and efficacy data. Overcoming these hurdles requires a paradigm shift toward integrated development strategies that balance therapeutic efficacy with manufacturability, safety, and regulatory compliance from early design stages. Ultimately, bridging the gap between compelling preclinical proof-of-concept and tangible patient benefit demands rigorous long-term safety evaluations (chronic toxicity, immunogenicity, and off-target effects) and well-designed, stratified clinical trials to validate these findings and ensure meaningful outcomes for NAFLD patients.

## Figures and Tables

**Table 1:** Search techniques tailored to databases for methodical literature retrieval.

Database	Complete Search Strategy
PubMed	{((“Nonalcoholic fatty liver disease”[Title/Abstract] OR NAFLD[Title/Abstract] OR NASH [Title/Abstract] OR “fatty liver”[Title/Abstract] OR “hepatic steatosis”[Title/Abstract] OR “liver fat accumulation”[Title/Abstract]) AND (nanoparticles[Title/Abstract] OR nanocarriers[Title/Abstract] OR nanomedicine[Title/Abstract] OR liposomes[Title/Abstract] OR “polymeric nanoparticles”[Title/Abstract] OR “solid lipid nanoparticles”[Title/Abstract] OR nanoformulations[Title/Abstract] OR “nano drug delivery”[Title/Abstract] OR “lipid nanoparticles”[Title/Abstract]) AND (“in vivo”[Title/Abstract] OR “in vitro”[Title/Abstract] OR mice[Title/Abstract] OR rat[Title/Abstract] OR “animal study”[Title/Abstract] OR “cell line”[Title/Abstract] OR “clinical study”[Title/Abstract] OR “human study”[Title/Abstract]))
Scopus	TITLE-ABS-KEY((“nonalcoholic fatty liver disease” OR NAFLD OR NASH OR “fatty liver” OR “hepatic steatosis” OR “liver fat accumulation”) AND (nanoparticles OR nanocarriers OR nanomedicine OR liposomes OR “polymeric nanoparticles” OR “solid lipid nanoparticles” OR nanoformulations OR “nano drug delivery” OR “lipid nanoparticles”) AND (“in vivo” OR “in vitro” OR mice OR rat OR “animal study” OR “cell line” OR “clinical study” OR “human study”))
Web of Science (Core collection)	TS=((“nonalcoholic fatty liver disease” OR NAFLD OR NASH OR “fatty liver” OR “hepatic steatosis” OR “liver fat accumulation”) AND (nanoparticles OR nanocarriers OR nanomedicine OR liposomes OR “polymeric nanoparticles” OR “solid lipid nanoparticles” OR nanoformulations OR “nano drug delivery” OR “lipid nanoparticles”) AND (“in vivo” OR “in vitro” OR mice OR rat OR “animal study” OR “cell line” OR “clinical study” OR “human study”))
Filters	Language: English; publication period: 1 January 2021 and 4 April 2026.

## Data Availability

The original contributions presented in this study are included in the article. Further inquiries can be directed to the corresponding authors.

## Supplementary Materials

The following supporting information can be downloaded at: https://www.mdpi.com/article/10.3390/cimb48090883/s1.

## Abbreviations

ABCA1	ATP-binding cassette subfamily A member-1
ACC	Acetyl-CoA carboxylase alpha
ACOX1	Acyl-CoA oxidase 1
AGE	Advanced glycation end-products
ALP	Alkaline phosphatase
ALT	Alanine aminotransferase
AMPK	Adenosine monophosphate-activated protein kinase
AOPP	Advanced oxidation protein products
AOX1	Aldehyde oxidase
AST	Aspartate aminotransferase
cAMP	Cyclic adenosine monophosphate
CAT	Catalase
CCl4	Tetrachloromethane
CD36	Cluster of differentiation 36
Cidea	Cell death inducing DFFA like effector a
COX-2	Cyclooxgynase-2.
CPT-1a	Carnitine palmitoyl transferase 1a.
CYP7A1	Cytochrome P450 7A1.
DIO2	Iodothyronine deiodinase 2
ECM	Extracellular matrix
FASN	Fatty acid synthases
FATP2	Fatty acid transporter protein 2
FFA	Free fatty acid
GGT	γ-Glutamyl transferase
Gli 1	Glioma-associated oncogene 1
GPX	Glutathione peroxidase
GSH-Px	Glutathione peroxidase
HFD	High-fat diet
HO-1	Hemeoxygense-1
HOMA–IR	Homeostasis model assessment of insulin resistance
HSCs	Hepatic stellate cells
IL-1β	Interleukin-1β
IL-6	Interleukin-6
INSR	Insulin receptor
IPITT	Intraperitoneal insulin tolerance test
Irs1	Insulin receptor substrate 1
KLF2	Kruppel- like Factor 2
LDH	Lactic dehydrogenase
LDL-c	Low-density lipoprotein cholesterol
NAFLD	Non-alcoholic fatty liver disease
NASH	Non-alcoholic steatohepatitis
NF-κB	Nuclear factor-kappa B
n-HDL-C	Non-high-density lipoprotein
NLRP3	Pyrin domain-containing 3.
NQO-1	Quinone oxidoreductase-1
Nrf2	Nuclear Factor Erythroid 2–Related Factor 2
MDA	Malondialdehyde
MPO	Myeloperoxidase
OGTT	Oral glucose tolerance test
PBS	Phosphate buffered saline
PCO	Plasma protein carbonyl
Pgc1α	Peroxisome proliferator-activated receptor gamma coactivator 1-alpha
PRDM16	PR domain containing 16
PTCH-1	Protein patched homolog 1
PTEN	Phosphatase and tensin homolog
Ptprf	Protein tyrosine phosphatase receptor type F
PPARα	Peroxisome proliferator activated receptor α
PPARγ	Peroxisome proliferator-activated receptor gamma
ROS	Reactive Oxygen Species
SCD1	Stearoyl-CoA desaturase 1
Slc2a2	Solute carrier family 2 member 2
SOCS3	Suppressor of cytokine signaling 3
SOD	Superoxide dismutase
SIRT1	Silent information regulating factor 1
SREBP-1c	Sterol regulatory element-binding protein-1c
α-SMA	α-smooth muscle actin
SMO	Smoothened co-receptor
TAC	Antioxidant capacity
TC	Total cholesterol
T2DM	Type 2 diabetes mellitus
TG	Triglycerides
TGF-β1	Transforming growth factor-β1
TRX	Thioredoxin
TrxR-1	Thioredoxin reductase-1
TK	Thioketal
TLR4	Toll-like receptor 4
TNF-α	Tumor necrosis factor-α
TXNIP	Thioredoxin-interacting protein
UCP1	Uncoupling protein-1
WAT	White adipose tissues
