# Supplementary material for: Liposomal Nanocarriers in Non-Alcoholic Fatty Liver Disease: A Systematic Review of Formulation Design, Targeting Strategies, and Therapeutic Outcomes
Source: Curr Issues Mol Biol. 2026 Aug 30;48(9):883. doi: 10.3390/cimb48090883 (PMC13605505; doi:10.3390/cimb48090883)
Supplement: Supplementary file 1 [file cimb-48-00883-s001.zip › cimb-4511149-supplementary.pdf]

Table S1. Liposomal Nanocarriers Examined for Experimental NAFLD/NASH studies: Colloidal and Physicochemical Characterization.

Platform	Particle Size (nm) /DLS	Polydispersity index (PDI)	Zeta Potential (ZP; mV)	Morphology/ Technique	Encapsulation Efficiency (EE)/ Drug Loading (DL)	Release Profile	Storage Condition / Stability	Ref
Dextran-coated multilayer oral bilosome (DEAE-DEX@SDBS)	150 nm	< 0.3	Changed after dextran coating and bilosome formation from +6 to ~0 mV *(approximate values based on published figures).*	Almost spherical with a fluffy outer layer/TEM	EE (BER~ 58-60 %; CUR~ 68-70 %); DL (BER~ 7.0 %; CUR~ 2.4 %); EE and DL reported as LR and LC, respectively; *(approximate values based on published data).*	BER 1st released at pH 1.2 at 1 h; from a gradual release at pH 6.8 to a total release of ~50 % at 24 h.<br>CUR continuously released with a cumulative release of < 20 % at 24 h.	Lyophilized powder, stored at 4°C/ NR	[18]
PEGylated vitexin liposomes (PEG-VLPs)	VLPs: 168 nm<br>PEG-VLPs: 458 nm	VLPs: 0.24<br>PEG-VLPs: 0.498	Changed after PEGylation from −9.6 to ~0 mV	Spherical (SEM)	EE= 94.18± 1.12 %; DL: NR	After 48 h: PEG-VLPs at 42 %; sustained cumulative drug release	NR	[20]
Adipose-targeted T3 liposome (PLT3)	NR	NR	Changed after PTP from −9.2 to +7.4 mV	Spherical (TEM)	NR	Continuous release: ~43 % of T3 after 8 h, and ~65 % after 72 h. Total release occurred after demulsification with acetonitrile	NR	[27]
Silybin liposome (SLB-Liposome)	119.76 nm	NR	-4.76 mV	Spherical (SEM)	EE: ~ 83-85 %; DL: NR	NR	NR	[14]
Simvastatin-loaded nanoliposome (SIM-LipoNPs)	SIM-LipoNPs: 139.5 nm	NR	-53 to -57 mV	Spherical (AFM)	NR	Sustained release: ~35 % after 72 h	Lyophilized product kept at −20 °C/ NR	[28]
Dasatinib/quercetin liposomes targeting white adipose tissue (D+Q liposomes)	145 nm	NR	-37.2 mV	Spherical (TEM)	EE: Q = 98 %; D = 97.2 %; DL: NR	Continuous release; over 80 % of D, and all Q released in ~ 50 h	NR	[21]
Dual-targeted sodium acetate liposomes (NaA@SC/MAN-LPs)	94.15 ± 0.23 nm	0.23± 0.01	+ 33 mV	Spherical (TEM)	EE= 76.6± 0.47 %; DL= 33.16± 0.16 %	Minimal release: serum <6 %, SGF <17 %, and SIF *(approximate values based on published data).*	Kept at 4 °C for 3 weeks/ negligible variations in particle size, PDI, and ZP	[22]

Mannose-modified and conventional platensimycin liposomes (M-Lip/PTM; Lip/PTM)	Lip/PTM: 135 ± 2.57 nm; M-Lip/PTM: 138 ± 3.46 nm	M-Lip/PTM: 0.18 ± 0.026; Lip/PTM: 0.21 ± 0.045	+ 13 to + 14 mV	Spherical (TEM)	EE= 38.2 ± 2.3 %; DL= 8.70 ± 0.31 %	Both formulations sustained release (70–80 %) of PTM; 90 % cumulative release at 24 h.	NR	[29,39]
Glucagon-modified T3 liposome (GCG-lipo/T3)	86.7 nm	0.176	- 24.6 mV	Spherical, with a fluffy peripheral bilayer (TEM)	EE= 82.9 ± 5.3 %; DL: NR	Sustained release: 75–82 %; 11.9–13.4 % released in the first 1 h	For short-term use (≤7 days), stored at 4 °C; for long-term storage, freeze-dried and stored at −20 °C; NR	[23]
Silibinin-loaded liposome (Sil-Lip)	< 130 nm	< 0.3	~ -2.5 mV *(approximate value based on published figures).*	Spherical (TEM)	EE ~ 76 %; DL: NR	A sustained release; ~35 % cumulative release at 24 h under SGF/SIF conditions *(approximate values based on published figures).*	NR	[16]
Chrysin-loaded nanoliposome (CH-NL)	121± 8 nm	0.22	- 37.9 mV	Spherical (TEM)	EE= 91.79 %; DL= 10.49 %	NR	NR	[24]
Lycopene-loaded liposome (Lip-Lyco)	250.2 ± 15.26 nm	0.31	- 6.4 mV	Spherical (TEM)	EE= 85.13± 3.78 %; DL: NR	NR	Stored at 4 °C until used/ NR	[30]
Chitosan-coated lutein liposomes (Lu-lip-LC, Lu-lip-HC)	Lu-lip: 263.4 ± 3.20 nm; Lu-lip-LC: 315.9 ± 5.40 nm; Lu-lip-HC: 338.7 ± 3.01 nm	0.229–0.295	Changed after chitosan coating from -37 to +27–29 mV	Spherical (TEM)	EE= 83.27 ± 0.39, 92.32± 0.67, and; 95.32 ± 0.57 % for Lu-lip, Lu-lip-LC, and Lu-lip-HC, respectively; DL:NR	The chitosan coating contributed to a reduction in lutein loss, (lutein retention rates of 12.74 ± 0.72, 11.33 ± 0.94, and 8.39 ± 0.67 %) after 16 h for Lu-lip, Lu-lip-LC, and Lu-lip-HC, respectively.	NR/ Stable for 21 days under different pH conditions; chitosan coating improved stability at pH 6–6.5	[19]
Hyaluronic acid-coated ROS-responsive liposomes (RLLs)	100 nm	0.234 (RLLs); 0.248 (LY@Lips)	−21.57 ± 2.45 (LY@Lips); −23.09 ± 0.53 (RLLs)	Spherical (TEM)	NR	NR	NR	[15]
Gum Arabic-coated anthocyanin liposomes (ACNs-LNP)	~ 99.4 nm (80.53–122 nm, depending on GA quantity)	0.46	+15 to +20 mV	Spherical (SEM)	EE ≈ 64–66 % (higher EE at GA > 1mg/mL *(approximate values based on published figures).*	NR	NR	[33]

Galactose-targeted cationic liposome (Gal-LNP-RSV)	RLLs: 125.47 ± 2.11 nm; LY@Lips: 85.02 ± 3.54 nm	< 0.15	NR	Spherical (TEM)	EE > 85 %; DL: NR	A sustained-release pattern, with a cumulative release of 70 % over approximately 36 h *(approximate values based on published figure).*	Kept at 4 °C; lyophilized with sucrose/trehalose for long-term preservation/NR	[31]
Spirulina peptide-loaded nanoliposomes/ chitosomes (SP-LP, SP-Ch-LP)	SP-LP: 144.83 ± 4.50 nm; SP-Ch-LP: 130.76 ± 2.34 nm	SP-LP: 0.41 ± 0.03; SP-Ch-LP: 0.32± 0.02	Changed after chitosan coating from -41 to +27 mV	Spherical (TEM+SEM)	NR	SP-Ch-LP: Biphasic sustained release = 22.86 % after 4 h, ~60.07 % after 24 h (PBS, pH 7.4), and 67.15 % during simulated GI digestion (4 h). SP-LP: Biphasic sustained release = 32.69 % at 4 h, and 75.21 % under simulated GI digestion (4 h).	Stable when stored at 4 °C; particle size, ZP, and antioxidant activity showed no significant changes. After 24 days, EE dropped by approximately 16.15 %; higher temperatures resulted in increases in particle size and PDI.	[32]
Aptamer-functionalized allicin liposome (LAA)	Lip-allicin: 62.19 ± 0.45 nm; Lip-allicin-aptamer: 96.34± 0.68 nm	Lip-allicin: 0.217 ± 0.016; Lip-allicin-aptamer: 0.198 ± 0.023	Shifted from near-neutral liposome to -34.7 mV after aptamer functionalization	Spherical (SEM)	EE: 59.20 and 61.58 %, for Lip-allicin and Lip-allicin-aptamer, respectively; DL: NR	NR	Stable at 4 °C for 4 weeks/ NR	[25]
Hybrid liposomal platform (Lip, Slip, C1-Lip, C1-Slip, Mlip)	122-134 nm	NR	-30 to -40 mV	Spherical (TEM)	EE >90 %; DL: 5.95-6.20 %	Sustained release of ~ 45-50 %; cumulative release over 24 h (PBS, pH 7.4)	Stable at 4 °C for ≥ 5 days, slight changes in particle size	[26]

Table S2. Targeting Techniques, Biodistribution, and Cellular Uptake in Experimental NAFLD and NASH

Active agent/ Cargo	Lipid Carrier	Functionalizatio/ Surface Modification	Targeting Strategy	*In vivo* and *in vitro* Models	Route of Administration/ Dose/ Duration	Biodistribution/ Cellular Uptake	Safety/ Biocompatibility	Ref
Berberine (BER) and Curcumin (CUR)	Soy phospholipid, Cholesterol, Sodium deoxycholate (bilosome)	None/lactibionic acid (LBA) modification, DEAE-dextran (DEAE-DEX) coating	Passive liver targeting via enhanced oral absorption and mediated hepatic uptake	*In vivo*: High-fat/high-sucrose diet-induced NAFLD in male C57BL/6J mice Kunming strain (male) for pharmacokinetics/biodistribution; i*n vitro:* Caco-2 cells	*In vivo*: BER/CUR (45/14 mg/kg), (oral gavage; OG), daily; 8 weeks; *in vitro:* BER/CUR (90/30 μg/mL); 2 h (permeability) or 24 h (cytotoxicity)	↑Oral bioavailability (BER: 34-fold; CUR:13-fold); ↑mucus and Caco-2 permeation >10-fold; ↑ liver biodistribution	↓Cytotoxicity against LO2 cells <10% inhibition at therapeutic concentration/ NR	[18]
Vitexin	DPPC/ Cholesterol	None/ PEG-6000 coating	Passive liver delivery *via* PEGylation to ↑stability, extended circulation and ↑oral delivery	*In vivo*: Female Sprague-Dawley rats with CCl4/urethane-induced NASH/NAFLD-like cirrhosis; *in vitro:* NA	*In vivo*: Vitexin: (10 mg/kg, IV or OG) VLPs (500 μg/kg, IV); PEG-VLPs: 500 μg/kg (OG), 15 days, following 6-weeks	NR	↑Histopathology of liver, kidney, and spleen/ NR	[20]
Triiodothyronine (T3)	Hydrogenated soybean phosphatidylcholine (HSPC), cholesterol and DSPE-PEG2000	Conjugation of the surface with an adipose-homing peptide (PTP)/ DSPE-PEG5K-maleimide	Active targeting of adipose tissue *via* PTP binding to prohibitin on the vasculature of adipose tissue	*In vivo*: HFD-induced obese C57BL/6N and $ApoE^{-/-}$ atherosclerotic mice; *in vitro*: Mouse adipocytes	*In vivo*: 50 μg/ kg T3 (intraperitonitally; IP); every 2 days for 32 days, biodistribution (8h); *in vitro*: 100 nM T3 for 8 days; uptake assay: 50 μmol/mL for 24 h	Selective accumulation in iWAT, eWAT, and iBAT; ↑adipocyte uptake and ↓off-target distribution.	Favorable *in vivo* safety; no toxicity in cardiac and skeletal muscles or endocrine system/ NR	[27]
Silybin (SLB)	Phospholipids and cholesterol	None	Passive liver targeting	*In vivo:* Male Sprague-Dawley rats with T2D-NAFLD caused by combination of HFD and STZ. *in vitro*: rat primary hepatocytes	*In vivo*: SLB equivalent (70 mg/kg/day, OG) for 4 weeks; *in vitro*: SLB equivalent (60 μM) for 24 h	NR/ ↑hepatocyte viability and glucose consumption *in vitro*	↑Liver function ↑improvement of hepatic histopathology.	[14]
Simvastatin (SIM)	Cholesterol and saturated phosphatidylcholine (DSPC)	None	Passive liver targeting	*In vivo*: NR*; in vitro*: human liver fibrotic microtissues (HepG2/LX-2/HUVECs)	*In vitro*: 5 or 10 mg/mL; 4 days after 4 days FFA induction	FITC-labelled liposomes showed qualitative cellular uptake	Non-toxic at concentrations of 5–10 mg/mL; ↓cell viability at 50 mg/mL	[28]
Dasatinib (D) and Quercetin (Q)	Egg yolk lecithin	P3 peptide/DSPE-PEG2000-P3 coating	P3-mediated WAT targeting (Putative)	*In vivo*: HFD-induced obese C57BL/6N and $ApoE^{-/-}$ atherosclerotic mice. *in vitro*: FAA-treated NCTC1469 hepatocytes and senescent adipose progenitor cells	*In vivo*:150 μL/ injection (IP) (3 injections over 1st week)-followed up for 4 weeks; *in vitro*: D+Q (1:10;1/10 μM) for 48 h.	↑Accumulation in WAT at 24 h/ NR	↓Toxicity to normal adipose tissue progenitor cells/ ↓Systematic hepatic toxicity	[21]

Sodium acetate (NaA)	Cholesterol, cationic cholesterol, PEGylated phospholipid and phosphatidylcholine	Mannose (MAN), sodium cholate (SC)/ DSPE-PEG2000-SC, DSPE-PEG2000-MAN	Dual active targeting: MAN-mediated Kupffer cell targeting *via* mannose receptor; SC-mediated intestinal absorption and hepatocyte targeting via ASBT/NTCP	*In vivo*: C57BL/6J mice with HFD-induced NAFLD; *in vitro*: H9c2 cells, Caco-2, FFA-induced AML-12, and Kupffer activated by LPS	*In vivo*: suspension 200 µL (25 mg/mL, OG), every other day for 10 weeks; *in vitro*: 2 mM NaA equivalent for 24h.	↑ Hepatic retention; ↑liver accumulation ↑intestinal, hepatocyte and Kupffer cell receptor-mediated uptake	Well tolerated *in vivo*, no systemic harm; good *in vivo* tolerance, low cytotoxicity (>85% cell viability)	[22]
Platensimycin (PTM)	Cholesterol and saturated phosphatidylcholine (DSPC)	Mannose (MAN) / DSPE-PEG2000-MAN coating	Active Kupffer cell targeting via MAN modification (Putative)	*In vivo*: NAFLD in C57BL/6J mice induced by Western diet and CCl_4; *in vitro*: HepG2 cells induced by FFA	*In vivo*: 10 mg/kg (IP) for 5 weeks; *in vitro*: HepG2 (5-50 µM, 48 h); cytotoxicity ≤ 500 µM	NR: Indirect evidence ↑Hepatic delivery	Treatment was well tolerated in mice (no hepatotoxicity)/no cytotoxicity toward HepG2 cells (5–500 µM).	[29,39]
Triiodothyronine (T3)	Soy phosphatidylcholine and cholesterol	Glucagon peptide (GCG)/ DSPE-PEG2000-GCG	Glucagon receptor-mediated liver targeting: ↑T3 delivery to the liver and adipose tissues, ↓heart and bone (Putative)	*In vivo*: ob/ob and C57BL/6 mice; *in vitro*: PBS/FBS release and stability tests	IP: 75 nmol/kg/day, 6-30 days; IV (single dose, biodistribution, 24 h)	↑Hepatic accumulation/ NR	No toxicity reported after 30 days /NR	[23]
Silibinin (Sil)	Saturated (DPPC) and unsaturated (POPC) phosphatidylcholine, cholesterol and cholic acid	None	Passive liver targeting by ↑oral absorption, mucus penetration, and GI stability	*In vivo*: HFD-induced C57BL/6 mice; *in vitro*: HepG2 cells induced with FFA; mucus penetration, cellular uptake, and release tests	*In vivo*: 50 mg/kg/day, intragastric (IG), 12 weeks, 4 weeks following HFD induction; *in vitro*: 20 µM Sil (24 h)	↑Oral bioavailability/ NR	No toxicity reported)/no cytotoxicity toward HepG2 cells (≤100 µM)	[16]
Chyrsin (CH)	Egg yolk lecithin and cholesterol	None	Passive liver delivery by ↑oral absorption, hepatic accumulation	*In vivo*: methionine and choline-deficient (MCD) diet-induced NASH in male C57BL/6J mice; *in vitro*: NR	*In vivo*: 100 mg/kg, IG, every other day for two weeks, MCD diet protocol (4 weeks); bioavailability study: 50 mg/kg three times, 6 h intervals	CH-NL ↑in liver and serum (2.04-fold and 3.32-fold, respectively), relative to free CH/ NR	CH-NL: no liver injury/ NR	[24]
Lycopene (Lyco)	Soy phosphatidylcholine and cholesterol	None	Passive oral delivery with ↑bioavailability and ↑hepatic efficacy	*In vivo*: Obese of NAFLD induced with HFD in male Sprague-Dawley rats; *in vitro*: NR	*In vivo*: 10 mg/kg/day, IG, for 8 weeks after 12-week HFD induction	NR	No hepatic anomalies/biocompatibility NR	[30]
Lutein	Soybean lecithin and cholesterol	None/coating with low or high molecular chitosan	Passive oral delivery, ↑bioavailability, cellular uptake and stability	*In vivo*: HFD-induced C57BL/6 mice; *in vitro*: uptake assay (HepG2/HUVEC); H_2O_2-	*In vivo*: Equivalent to 50 mg lutein/kg, OG, 8-week treatment; *in vitro*: 16 µM lutein equivalent/24h	No direst evaluation, ↑GI bioavailability Lu-lip-HC/↑ cellular	No detectable toxicity under tested conditions/ No HepG2 toxicity	[19]

				induced oxidative stress in HepG2		uptake of Lu-lip-HC *in vitro*		
PI3K inhibitor LY294002 + oridonin	DOPC, cholesterol and DSPE-PEG2000	Hyaluronic acid (HA) / HA–thioketal–oridonin coating	CD44-mediated hepatic stellate cell targeting with ROS-responsive drug release	*In vivo*: CCl_4-induced NAFLD/ fibrosis in male Balb/C mice; *in vitro*: palmitic acid-induced AML-12 hepatocytes and LX-2 hepatic stellate cells	*In vivo*: LY294002 (10 mg/kg) + oridonin (5 mg/kg); (IV, every 3 days for 15 days*); in vitro*: PA 100 µM; LY294002 (0.4-500 µM) + oridonin (0.08-100 µM) for 24 h	↑ hepatic accumulation/ ↑*in vitro* cellular uptake via HA-CD44	No significant cytotoxicity reported, LY294002 ≤ 500 µM, oridonin ≤ 100 µM	[15]
Blueberry anthocyanin (ACNs)	Soy lecithin	None/gum Arabic (Lipo-GA) coating	Passive oral liver-targeted delivery	*In vivo*: HFD-induced obese male C57BL/ 6J mice; *in vitro*: oleic acid-induced HepG2 steatosis and oxidative stress model	*In vivo*: 615 mg/kg/day ACNs-equivalent (OG), 10 weeks; *in vitro* (40µg/ mL; biocompatibility study)	NR/ ↑HepG2 uptake	No adverse effects on morphology or viability at 40µg/mL	[33]
Resveratrol (RSV)	Ionizable cationic lipid (MC3), DSPC, cholesterol, DMG-PEG2000	Galactose (Gal)/ Gal-PEG2000-DSPE	Asialoglycoprotein receptor (ASGPR)-mediated active hepatocyte targeting	*In vivo*: HFD-induced female BALB/c mice; *in vitro*: OA/P-induced HepG2 steatosis	*In vivo*: 100 mg/kg RSV-equivalent (IV) every 3 days (6 weeks), biodistribution study (5mg/kg, 2-8h); *in vitro*: (1-10 µM, 24h)	3.9-fold ↑liver accumulation/ 3.5-fold ↑ASGPR-mediated HepG2 uptake	Treatment-related toxicity (NR)/ non-toxic (HepG2 ≤1 mg/mL)	[31]
Spirulina peptides	Soy/lecithin/ cholesterol/ Tween 80	None/Chitosan coating (SP-Ch-LP)	Passive oral delivery	*In vivo*: HFD-induced NAFLD in male Wistar rats; *in vitro*: L-929 cytotoxicity assay	*In vivo*: 200 mg/kg/day (orally) for 4 weeks after 8 weeks HFD induction; *in vitro*: 5-100 µg/ml (24, 48, 72 h)	NR/sustained GI release for SP-Ch-LP	Non- toxic (5-100 ug/ml); SP-LP and SP-Ch-LP showed good biocompatibility	[32]
Allicin	DMPC, cholesterol, DSPE-PEG2000	NAFLD01 aptamer/ cholesterol-modified NAFLD01 aptamer	Active targeting CD36-overexpressing NAFLD cells via NAFLD01 aptamer (Putative)	*In vivo*: HFD-induced NAFLD/ DIO male C57BL/ 6J mice; *in vitro:* OA-induced HepG2 steatosis model	*In vivo*: 10/20 mg/kg allicin, IP, 5 weeks after 10-weeks HFD induction: *in vitro:* cytotoxicity: 0-650 µM, 24h; uptake assay 4 h	NR/ ↑uptake in NAFLD HepG2 (higher than in normal cells)	Non- toxic/ good HepG2 biocompatibility	[25]
Silibinin and surface collagenase I (C1)	Egg phosphatidyl choline (EPC) and cholesterol and stearylamine	CD47-drived self-peptide surface/ self-peptide conjugation, collagenase I surface adsorption (C1-Lip)	Immune-evasive liver delivery, CD47-SIRPα signaling; ↓Kupffer cell clearance and ↑hepatocyte/ parenchymal delivery (Putative)	*In vivo*: Lep ob/ob male mice with HFD-induced NAFLD/ fibrosis; BALB/c mice for biodistribution; *in vitro:* LX-2 hepatic stellate cells, RAW264.7 macrophages	*In vivo*: 3 mg/kg silibinin equivalent and collagenase I (0.5 mg/kg equivalent), weekly (4 weeks); *in vitro*: 50 µg/mL equivalent, 24 h	↑Hepatic retention, ↓Kupffer clarence/ ↑uptake by hepatocytes and endothelial cells	↑improvement in liver function/ no macrophage polarization induced	[26]

Table S3. Molecular mechanisms underlying the therapeutic effects of liposomal-based nanomedicines in NAFLD.

Platform	*In vivo* and *in vitro* models	Therapeutic effect	Mechanism	Key finding	Ref
Dextran-coated multilayer oral bilosome (DEAE-DEX@SDBS)	*In vivo*: high-fat/high-sucrose diet-induced NAFLD in male C57BL/6J, mice Kunming strain (male) for pharmacokinetics /biodistribution; *in vitro:* Caco-2 cells	Amelioration of NAFLD by reducing hepatic lipid accumulation, inflammation, oxidative stress, dyslipidemia, liver injury, fat accumulation, and fasting serum glucose levels	Activated Nrf2 signaling *via* Nrf2 nuclear translocation and upregulated NQO-1, HO-1, and TrxR-1. Inhibited NF-κB signaling by suppressing phosphorylation of p65 and IκBα. suppressed NLRP3 inflammasome activation by ↓NLRP3, Caspase-1, IL-6. Promoted TXNIP-TRX and p300-Nrf2 interaction while ↓TXNIP-NLRP3 and p300-p65 interactions, resulting in enhanced antioxidant and anti-inflammatory effects	Improved liver histopathology, ↓TC, TG, LDL-c, AST, ALT, ALP, GGT, LDH, and MDA levels, ↑SOD and GSH levels, and showed the strongest therapeutic efficacy among all tested formulations	[18]
PEGylated vitexin liposomes (PEG-VLPs)	*In vivo*: Female Sprague-Dawley rats with CCl4/urethane - induced NASH/NAFLD-like cirrhosis; *in vitro:* NA	Reduced NASH/NAFLD-related cirrhosis manifestations by improving liver histopathology, liver function indices, body/liver weight changes, and ascites; PEG-VLPs showed the best therapeutic response	Vitexin activation of lipolysis and fatty acid oxidation, antioxidant/hepatoprotective activity, and antifibrotic effects.	In treated rats, PEG-VLPs oral gavage showed normal liver histology, with prominent Kupffer cells and proliferating hepatocytes; improved serological indices, ↓weight reduction, and absence of ascites, relative to diseased controls	[20]
Adipose-targeted T3 liposome (PLT3)	*In vivo*: HFD-induced obese C57BL/6N and ApoE^{-}/$^{-}$ atherosclerotic mice; *in vitro*: Mouse adipocytes	Ameliorated obesity-associated metabolic dysfunction including obesity, glucose intolerance insulin resistance, fatty liver, hypercholesterolemia, and atherosclerosis	Selective enrichment of T3 in adipose tissue induced browning and thermogenesis of WAT, enhanced energy expenditure, ↓adipose inflammation, ↑adiponectin production, improved insulin sensitivity, ↓hepatic lipid accumulation, and ↓circulating cholesterol. PLT3 prevented the feedback suppression of sympathetic innervation activity observed with T3 therapy, thereby maintaining adrenergic simulation of adipose tissue.	PLT3 produced greater reductions in body weight gain, fat mass adipocyte size, glucose intolerance, insulin resistance, fasting insulin, hepatic steatosis, hepatic TG, FFA, ALT, AST, serum TC and LDL-c, and atherosclerosis lesion area, than free T3 or non-targeted liposomal T3 (LT3). PLT3 markedly ↑WAT browning pathway (↑Ucp1, ↑Dio2, ↑Pgc1α, ↑Cidea, ↑Prdm16) and thermogenic pathway (↑UCP1, ↑oxygen consumption), ↓lipid accumulation in white adipocytes, and circulating cholesterol; sympathetic signaling pathway (↑norepinephrine, ↑cAMP, ↑FFA); adipose inflammatory pathway (↓TNF-α,↓IL-1β, ↓IL-6, ↓Mcp1, ↓F4/80 macrophages), and adiponectin pathway (↑adiponectin)	[27]
Silybin liposome (SLB-Liposome)	*In vivo:* Male Sprague-Dawley rats with T2D-NAFLD caused by combination of HFD and STZ;	Ameliorated T2DM-NAFLD by decreasing insulin resistance, glucose metabolism, lipid metabolism, inflammation, fibrosis, and liver dysfunction	Activated AMPK signaling and inhibited TGF-β1/Smad2/3 phosphorylation; ↓ECM deposition and fibrogenesis *via* ↓collagen I, collagen III, and α-SMA expressions; improved hepatocyte viability and glucose consumption.	Liposomal SLB ↓fasting glucose, improved OGTT and IPITT, ↑ HDL-C, ↓TC and TG, ↓AST and ALT, alleviated hepatic lipid accumulation, inflammation, fibrosis, and NAFLD activity score; ↓serum TNF-α, IL-1β, and IL-6.	[14]

	in vitro: rat primary hepatocytes			Liposomal SLB produced stronger therapeutic effects than free SLB,	
Simvastatin-loaded nanoliposome (SIM-LipoNPs)	*In vivo*: NR; *in vitro*: human liver fibrosis microtissues (HepG2/LX-2/HUVECs)	Attenuated NAFLD- associated liver fibrosis by reducing oxidaive stress, inflammation, collagen deposition, and fibrotic progression	Activated the SIM-KLF2-NO signaling axis, resulting in ↑KLF2 expression and NO production. ↓oxidative stress through suppression of ROS generation; suppressed inflammatory response by downregulating TNF-α, IL-1, IL-1β, and IL-6; inhibited HSC activation and ECM accumulation through reduction of collagen I expression	SIM-LipoNPs ↑KLF2 and NO levels, ↓ROS production, ↓pro-inflammatory cytokine expression, ↓collagen I mRNA, improved cell viability, and anti-fibrotic efficacy more than free SIM. Notably, 5 mg/mL SIM-LipoNPs produced effects comparable to, or better than higher doses of free SIM	[28]
Dasatinib /Quercetin liposomes targeting white adipose tissue (D+Q liposomes)	*In vivo*: HFD-induced obese C57BL/6N and $ApoE^{-/-}$ atherosclerotic mice; *In vitro*: FAA-treated NCTC1469 hepatocytes and senescent adipose progenitor cells	Alleviated age-related hepatic steatosis and hepatocyte senescence *via* ↓ WAT lipolysis and ↓FFA influx into the liver	Targeted elimination of senescent cells in WAT, ↓senescent markers (SA-β-gal, p16, p21, p53 and SASP factors); suppressed lipolysis by downregulating PNPLA2/ATGL, Mgll and Lipe; ↓circulating FFAs, ↓hepatic lipid accumulation and attenuated hepatocyte senescence	Aging ↑WAT senescence, lipolysis, circulating FFA levels, hepatic TG and FFA accumulation, hepatic steatosis, and hepatocyte senescence. WAT-targeted D+Q liposomes eliminated senescent cells in WAT more effectively than conventional D+Q administration, ↓circulating FFAs, ↓lipid accumulation in the liver, improved hepatic lipid metabolism genes (CPT1α, Ucp2, Accα, Acox1, CD36, and Scd1), and markedly ↓hepatocyte senescence markers.	[21]
Dual-targeted sodium acetate liposomes (NaA@SC/MAN-LPs)	*In vivo*: C57BL/6J mice with HFD-induced NAFLD; *in vitro*: H9c2 cells, Caco-2, FFA-induced AML-12, and Kupffer activated by LPS	Alleviated NAFLD by ↓hepatic lipid accumulation, improving lipid metabolism, suppressing Kupffer cell-mediated inflammation, improving glucose tolerance, and attenuating liver injury	Enhanced hepatic delivery and retention of sodium acetate, activated AMPK signaling in hepatocytes, ↓lipid accumulation, promoted FA oxidation, suppressed macrophage infiltration, ↓NF-κB activation, ↓pro-inflammatory cytokine expression, and alleviated inflammatory responses	Liposomes significantly ↓TG, TC, lipid droplets, and lipogenic gene expression while ↑CPT1α. In Kupffer cells, liposomes ↓TNF-α, IL-1β, and IL-6 and ↑IL-4. In NAFLD mice, NaA@SC/MAN-LPs ↓body weight gain, improved OGTT, ↓serum TG, TC, LDL-C, ALT, and AST; ↓hepatic TG, TC, lipid droplets area, macrophage infiltration, and hepatic inflammation more effectively than free NaA.	[22]
Mannose-modified and conventional platensimycin liposomes (M-Lip/PTM)	*In vivo*: NAFLD in C57BL/6J mice induced by Western diet and CCl_4; *in vitro*: HepG2 cells induced by FFA	Attenuated NAFLD progression by ↓hepatic steatosis, ↓hepatic TG accumulation, dysregulated glucose metabolism, and ↓*de novo* lipogenesis	Inhibited FASN, resulting in suppression of *de novo* lipogenesis. Downregulated lipogenic regulators and the enzymes SERBP-1c, ACC, and SCD1 while enhancing lipid oxidation-associated genes (PPARα and ACOX1); ↓hepatic droplet formation and hepatic TG accumulation.	PTM ↓hepatic steatosis, ↓hepatic TG accumulation, fasting blood glucose, and improved glucose tolerance in mice. PTM ↓FASN protein expression in mouse liver and FFA-treated HepG2 cells. PTM ↓expression of FASN, SERBP-1c, ACC, and SCD1 while ↑expressions of PPARα and ACOX1. M-Lip/PTM strongest ↓hepatic TG and lipid droplet accumulation to normal levels.	[29]
Glucagon-modified T3 liposome (GCG-lipo/T3)	*In vivo*: ob/ob and C57BL/6 mice; *in vitro*: PBS/FBS release and stability tests	↓Obesity-associated hepatic steatosis, dyslipidemia, and hyperglycemia by ↓body weight, ↓hepatic lipid accumulation, and improving metabolic homeostasis	Targeted hepatic and adipose delivery of T3 enhanced energy expenditure, promoted fat, ↓induced browning of adipose tissue through UCP1 upregulation, ↓hepatic lipid	↓Body weight, ↓hepatic lipid droplets and steatosis; improved plasma TG, TC, LDL, HDL, ↓FFA and hepatic lipase, improved glucose tolerance and insulin sensitivity, ↓adipocyte size, and ↑UCP1 expression in	[23]

			accumulation, improved glucose tolerance, improved insulin sensitivity, and corrected dyslipidemia while preventing systemic thyrotoxicity	inguinal and perirenal adipose tissue. No significant cardiovascular, bone, kidney, pancreatic, or thyroid toxicity was observed.	
Silibinin-loaded liposome (Sil-Lip)	*In vivo*: HFD-induced C57BL/6 mice; *in vitro*: HepG2 cells induced by FFA, (mucus penetration, cellular uptake, and release tests)	Ameliorated NAFLD by reducing hepatic steatosis, lipid metabolism disorders, insulin resistance, and inflammation	Suppressed hepatic insulin resistance, inhibited NF-κB-mediated inflammatory responses, ↓macrophage infiltration, inhibited lipogenesis and FFA uptake, promoted lipolysis, and restored gut microbiota dysbiosis	In NAFLD mice, Sil-Lip ↓body weight gain, liver weight, liver index, hepatic steatosis, collagen deposition, AST, ALT, LDL-c, TC, TG, FFA, fasting insulin, HOMA-IR, TNF-α, IL-1β, IL-6, F4/80, and CD68-postive macrophage infiltration. Sil-Lip normalized SREBP-1, CD36, FATP2, and FASN expression. In HepG2 cells, Sil-Lip ↓intracellular TG accumulation, improved glucose uptake, ↓inflammatory cytokines, and reversed abnormal lipid metabolism-related proteins. Moreover, Sil-Lip restored gut microbiota diversity and composition more effectively than free Sil.	[16]
Chrysin-loaded nanoliposome (CH-NL)	*In vivo*: methionine and choline-deficient (MCD) diet-induced NASH in male C57BL/6J mice; *in vitro*: NR	Ameliorated NASH by ↓hepatic steatosis, liver injury, oxidative stress, inflammation, and fibrosis	Inhibited TLR4 signaling pathway activation, resulting in suppression of p38 MAPK and NF-κB signaling (↓ p65); ↓inflammatory cell infiltration, oxidative stress, and fibrogenesis; ↓ROS generation and MPO expression, ↓macrophage and neutrophil infiltration, and suppressed collagen deposition	CH-NL ↓AST, ALT, hepatic TG accumulation, NAFLD activity score, ROS production, MPO expression, CD45+ leukocyte infiltration, F4/80 + macrophage infiltration, neutrophil infiltration, TNF-α, IL-6 expression, collagen deposition, fibrosis score, α-SMA expression, and inhibited protein expressions of TLR4, p38, and p65. CH-NL consistently produced stronger effects than free CH.	[24]
Lycopene-loaded liposome (Lip-Lyco)	*In vivo*: Obese of NAFLD induced by HFD in male Sprague-Dawley rats; *in vitro*: NR	Alleviated NAFLD by reducing obesity, insulin resistance, hepatic steatosis, dyslipidemia, oxidative stress, inflammation, apoptosis, and impaired autophagy	↓Obesity-associated metabolic dysfunction, improved insulin sensitivity, restored adipokine, inhibited apoptosis, activated autophagy, and inhibited Hedgehog signaling. Lip-Lyco upregulated AMPK, Becilin-1, LC3-II, Hhip, and PTCH-1 while downregulating mTOR, p62, SMO, PTCH-1, and Gli-1; ↓COX-2 expression and restored BCL-2 expression.	HFD-induced NAFL rats showed insulin resistance, dyslipidemia, oxidative stress, inflammation, apoptosis, impaired autophagy, and activated Hedgehog signaling. Lip-Lyco ↓body weight gain, liver weight, glucose, insulin, HOMA-IR, leptin, TG, TC, LDL-c, ALT, AST, TNF-α, IL-1β, IL-6, and MDA while ↑adiponectin, HDL-c, and BCL-2. Histologically, Lip-Lyco ↓hepatic steatosis and inflammatory infiltration and improved liver architecture	[30]
Chitosan-coated lutein liposomes (Lu-lip-LC, Lu-lip-HC)	*In vivo*: HFD-induced C57BL/6 mice; *in vitro*: uptake assay (HepG2/HUVEC) ; H_2O_2-induced	Reduced hepatic accumulation, lipid accumulation, obesity, dyslipidemia, oxidative stress and NAFLD progression	Enhanced cellular uptake of lutein, ↓ROS generation, improved mitochondrial membrane potential, ↑antioxidant enzyme activities (CAT, SOD, and GSH-Px); ↓MDA, ↓hepatic and adipose fat accumulation, and improved serum lipid profiles.	Lu-lip-HC ↓ROS level, apoapsis/necrosis, mitochondrial dysfunction, and oxidative damage while ↑CAT, SOD, and GSH-Px activities. In HFD-fed mice, Lu-lip-LC and Lu-lip-HC ↓body weight gain, liver weight, epididymal fat accumulation, serum TG, TC, n-HDL-C, and hepatic	[19]

	oxidative stress in HepG2			MDA, while ↑HDL-C and hepatic CAT, SOD, and GSH-Px activities. Histologically, both formulations ↓lipid vacuoles and hepatic lipid vacuoles and hepatic fat accumulation	
Hyaluronic acid-coated ROS-responsive liposomes (RLLs)	*In vivo*: CCl_4-induced NAFLD/ fibrosis in male Balb/C mice; *in vitro*: palmitic acid-induced AML-12 hepatocytes and LX-2 hepatic stellate cells	Ameliorated NAFLD by improving insulin resistance, dysregulating lipid metabolism, oxidative stress, inflammation, hepatic steatosis, and fibrosis	ROS-responsive thioketal linker scavenged excessive ROS and triggered controlled release of LY294002 and oridonin. RLLs maintained insulin sensitivity, ↓inflammatory signaling, attenuated oxidative stress, improved CYP3A11 activity, ↓lipid accumulation, suppressed fibrosis progression, and alleviated metabolic dysfunction.	In CCl_4 induced NAFLD mice, RLLs ↓NAFLD activity score, fibrosis score, collagen deposition, hepatocyte ballooning, lobular inflammation, lipid droplet accumulation, TG, cholesterol, TNF-α, IL-6, Collagen IV, ALT, and AST. RLLs improved SOCS3 3-fold and restored IRS1, INSR, PTEN, SOD, Catalase, and GSH-Px levels while suppressing NF-κB and TGF-β signaling.	[15]
Gum Arabic-coated anthocyanin liposomes (ACNs-LNP)	*In vivo*: HFD-induced obese male C57BL/ 6J mice; *in vitro*: oleic acid-induced HepG2 steatosis and oxidative stress model	Reduced hepatic lipid droplet accumulation, obesity-associated dyslipidemia, liver injury, oxidative stress, and gut microbiota dysbiosis	↓Intracellular lipid droplet accumulation and ROS generation, improved serum lipid metabolism, and modulated gut microbiota composition by ↑beneficial bacteria and ↓obesity-associated microbial alterations	In HepG2 cells, ACNs-LNP ↓Nile Blue A-stained lipid droplet and intracellular ROS more effectively than free ACNs. In HFD-fed mice, ACNs-LNP ↓body weight gain, serum TG, TC, LDL-C, AST, and ALT while ↑HDL-C. Histological analysis showed ↓hepatic lipid vacuoles and almost restored normal liver architecture. ACNs-LNP corrected HFD-induced gut microbiota imbalance more effectively than free ACNs.	[33]
Galactose-targeted cationic liposome (Gal-LNP-RSV)	*In vivo*: HFD-induced female BALB/c mice; *in vitro*: OA/P-induced HepG2 steatosis	Ameliorated hepatic steatosis by ↓hepatic lipid accumulation, decreasing dyslipidemia, restoring liver marker levels, and alleviating oxidative stress	↑Intracellular RSV activated lipid metabolism and antioxidants, resulting in ↓lipid deposition, improved redox hemostasis, and restoration of metabolic balance.	In HepG2 cells, LNP-RSV ↓Oil red O-positive lipid accumulation, intracellular TG, and TC more effectively than free RSV. In HFD-induced NAFLD mice, Gal-LNP-RSV ↓serum TG, LDL-C, ALT, AST, liver weigh ratio, hepatic lipid accumulation, hepatic TG, hepatic TC, and MDA levels while ↑HDL-C and SOD activity. Histological analyses demonstrated marked reduction in hepatic steatosis and improved liver architecture.	[31]
Spirulina peptide-loaded nanoliposomes/chitosomes (SP-LP, SP-Ch-LP)	*In vivo*: HFD-induced NAFLD in male Wistar rats; *in vitro*: L-929 cytotoxicity assay	Alleviated NAFLD by ↓hepatic lipid accumulation, steatosis, oxidative stress, inflammation, dyslipidemia, insulin resistance, and liver injury	Activated AMPK signaling and ↑AMPK phosphorylation, leading to inhibition of lipogenesis and stimulation of FA β-oxidation. Downregulated SREBP-1c, FASN, and ACCα while upregulating PPARα, CPT-1, AOX1, SIRT1, ABCA1, and CYP7A1; enhanced antioxidant defense (↑SOD, ↑GPX, ↓MDA) and suppressed inflammatory cytokines (↓TNF-α, ↓IL-1β, ↓IL-6). ↑p-ACC/ACC	In NAFLD rats, SP-LP and particularly SP-Ch-LP ↓body weight gain, liver weight, hepatic TG and TC accumulation, serum TG, TC, LDL, ALT, and AST; fasting glucose, HOMA-IR, oxidative stress markers (PCO, AGE, AOPP, MDA), and inflammatory cytokines. Histopathology showed ↓in hepatic lipid vacuoles, inflammatory infiltration, ballooning degeneration, and fibrosis. SP-	[32]

			ratio, thereby ↓FA synthesis and promoting lipid catabolism	Ch-LP generally produced stronger effects than free SPs.	
Aptamer-functionalized allicin liposome (LAA)	*In vivo*: HFD-induced NAFLD/ DIO male C57BL/ 6J mice; *in vitro:* OA-induced HepG2 steatosis model	Alleviated NAFLD by ↓hepatic lipid accumulation, hepatic steatosis, dyslipidemia, liver injury and abnormal lipid metabolism	Suppressed *de novo* lipogenesis by downregulation of ACC, FASN, SCD-1, SREBP1-c, PPARγ; ↓CD36 expression, thereby limiting FA uptake and hepatic lipid accumulation; improved systemic and hepatic lipid metabolism and attenuated liver injury.	In OA-treated HepG2 cells, LAA ↓body weight gain, liver index, serum TC, TG, LDL, AST, ALT, hepatic TC and TG accumulation, hepatic steatosis, lipid vacuole formation, and liver injury. Histology demonstrated marked ↓in hepatic lipid deposition. LAA consistently outperformed free allicin	[25]
Hybrid liposomal platform (Lip, Slip, C1-Lip, C1-Slip, Mlip)	*In vivo*: Lep ob/ob male mice with HFD-induced NAFLD/ fibrosis; BALB/c mice for biodistribution; *in vitro:* LX-2 hepatic stellate cells, RAW264.7 macrophages	Ameliorated hepatic steatosis, liver fibrosis, inflammation, dyslipidemia, and liver injury while restoring metabolic homeostasis	CD47-derived self-peptide (Slip) engaged the SIRPα pathway on Kupffer cells, suppressing phagocytosis and prolonging hepatic retention. Mixed liposomes enhanced delivery of silibinin to hepatocytes and HSC. Surface-bound collagenase I degraded ECM and improved tissue penetration. Silibinin suppressed inflammatory macrophage activation, ↓ROS generation, ↓IL-1β, IL-6, nitrite production, inhibited HSC activation, and ↓collagen synthesis and fibrosis.	C1- Mlip/silibinin ↓hepatic lipid liver accumulation, Sirius Red-positive fibrosis, collagen I deposition, F4/80-positive macrophage infiltration, serum TG, TC, ALT, AST, and hepatic collagen content; ↑circulating adiponectin and restored liver histology close to control levels. 3D liver imaging confirmed substantial ↓in lipid deposition. C1-Mlip/silibinin consistently outperformed C1- Slip/silibinin and conventional liposomes.	[26]
